# No Evidence of Plasmodium falciparum *k13* Artemisinin Resistance-Conferring Mutations over a 24-Year Analysis in Coastal Kenya but a Near Complete Reversion to Chloroquine-Sensitive Parasites

**DOI:** 10.1128/AAC.01067-19

**Published:** 2019-11-21

**Authors:** Kevin Wamae, Dorcas Okanda, Leonard Ndwiga, Victor Osoti, Kelvin M. Kimenyi, Abdirahman I. Abdi, Philip Bejon, Colin Sutherland, Lynette Isabella Ochola-Oyier

**Affiliations:** a KEMRI-Wellcome Trust Research Programme, CGMRC, Kilifi, Kenya; b Centre for Biotechnology and Bioinformatics, University of Nairobi, Nairobi, Kenya; c Pwani University Bioscience Research Centre, Pwani University, Kilifi, Kenya; d Nuffield Department of Medicine, Centre for Clinical Vaccinology and Tropical Medicine, Churchill Hospital, University of Oxford, Oxford, United Kingdom; e Department of Immunology and Infection, Faculty of Infectious Diseases, London School of Hygiene and Tropical Medicine, London, United Kingdom; f PHE Malaria Reference Laboratory, London School of Hygiene and Tropical Medicine, London, United Kingdom

**Keywords:** *Plasmodium falciparum*, artemisinin resistance, chloroquine resistance, *k13*, sulfadoxine-pyrimethamine resistance

## Abstract

Antimalarial drug resistance is a substantial impediment to malaria control. The spread of resistance has been described using genetic markers, which are important epidemiological tools. We carried out a temporal analysis of changes in allele frequencies of 12 drug resistance markers over 2 decades of changing antimalarial drug policy in Kenya. We did not detect any of the validated kelch 13 (*k13*) artemisinin resistance markers; nonetheless, a single *k13* allele, K189T, was maintained at a stable high frequency (>10%) over time. There was a distinct shift from chloroquine-resistant transporter (*crt*)-76, multidrug-resistant gene 1 (*mdr1*)-86 and *mdr1*-1246 chloroquine (CQ) resistance alleles to a 99% prevalence of CQ-sensitive alleles in the population, following the withdrawal of CQ from routine use. In contrast, the dihydropteroate synthetase (*dhps*) double mutant (437G and 540E) associated with sulfadoxine-pyrimethamine (SP) resistance was maintained at a high frequency (>75%), after a change from SP to artemisinin combination therapies (ACTs). The novel cysteine desulfurase (*nfs*) K65 allele, implicated in resistance to lumefantrine in a West African study, showed a gradual significant decline in allele frequency pre- and post-ACT introduction (from 38% to 20%), suggesting evidence of directional selection in Kenya, potentially not due to lumefantrine. The high frequency of CQ-sensitive parasites circulating in the population suggests that the reintroduction of CQ in combination therapy for the treatment of malaria can be considered in the future. However, the risk of a reemergence of CQ-resistant parasites circulating below detectable levels or being reintroduced from other regions remains.

## Abstract (precis)

Antimalarial drug resistance is a substantial impediment to malaria control. The spread of resistance has been described using genetic markers, which are important epidemiological tools. We carried out a temporal analysis of changes in allele frequencies of 12 drug resistance markers over 2 decades of changing antimalarial drug policy in Kenya.

## INTRODUCTION

Artemisinin-based combination therapies (ACTs) are recommended as the first-line treatment for uncomplicated Plasmodium falciparum malaria in all regions of endemicity; however, drug resistance is an impediment to malaria control. Notably, resistance to former first-line antimalarials, chloroquine (CQ) and sulfadoxine-pyrimethamine (SP), was imported to Africa via strains from South East (SE) Asia (1–3), although some drug resistance markers to pyrimethamine emerged independently in Africa (4) and were associated with increased malaria-related mortality in sub-Saharan Africa (5, 6). For this reason, the recent emergence and spread of artemisinin resistance in SE Asia (7–9), as well as of multidrug-resistant (resistance to both artemisinin and the partner drug, piperaquine) P. falciparum parasites in Cambodia and neighboring Vietnam (10, 11), are now a major concern. Though isolated, the recent possible identification of parasites with high artemisinin survival rates in Equatorial Guinea and Uganda (12, 13) calls for continued surveillance of artemisinin resistance markers in Africa.

The identification of P. falciparum genetic mutations associated with antimalarial drug resistance has provided molecular markers for surveillance of resistance, both in real time and retrospectively, to assess geographic origins and migration patterns of drug-resistant parasites (14). The use of such markers provided information on the origin and spread of CQ (1) and sulfadoxine-pyrimethamine (SP) (2, 3). Likewise, mutations in the kelch13 gene (*k13*), including F446I, N458Y, M476I, Y493H, R539T, I543T, P553L, R561H, and C580Y, are enabling the detection and tracking of artemisinin resistance in SE Asia (9, 15). Some of these mutations (Y493H, P553L, R561H, and C580Y) have been observed at low frequencies (<0.1%) in Africa (16). Whole-genome studies of artemisinin-resistant parasites in SE Asia were shown to accumulate artemisinin resistance predisposing mutations at other genetic loci (17). These mutations were found in apicoplast ribosomal protein S10 precursor gene (*arps10*) codon V127M, chloroquine resistance transporter (*crt*) codon I356T, ferredoxin (*fd*) codon D193Y, and multidrug resistance (*mdr*) protein 2 codon T484I. While these mutations have been identified in parasites from Africa, they occur at very low frequencies (*arps*-10-V127M, 0%; *fd*-D193Y, 0.1%; *mdr2*-T484I, 0.1%; *crt*-N326S, 0.8%) (16). Nonetheless, it is crucial for regions outside SE Asia to monitor the emergence of artemisinin resistance signatures, including the selection of markers associated with changes in antimalarial drug policy, drug trials, and experimental analyses. Already, recent studies have shown that artemether-lumefantrine (AL) selects for polymorphisms in the chloroquine resistance transporter (*crt*) (K76 allele) and multidrug resistance 1 (*mdr1*) (N86/184F/D1246-NFD haplotype) genes (18–21). Furthermore, the *mdr1*-NFD haplotype and an increase in *mdr1* copy number have been linked with reduced susceptibility to lumefantrine (LM) and mefloquine (MQ), respectively (22–24). Moreover, several recent studies have shown the selection of additional polymorphisms associated with ACT pressure and warrant further investigation. A mutation (S69Stop) in the cysteine proteinase *falcipain-2a* gene has previously been selected for after artemisinin *in vitro* selection pressure, using a parasite isolate from Africa (15). A clinical trial conducted in western Kenya revealed that 2 to 3 days of ACT treatment selected for parasites with either the 160N or 160T allele in the AP-2 complex subunit mu gene (*ap2-mu*, S160N/T) and the allele 1528D in the ubiquitin carboxyl-terminal hydrolase 1 gene (*ubp-1*, E1528D) (21). In The Gambia, a temporal increase in the frequency of the K65 allele (K65Q) in the cysteine desulfurase (*nfs*) gene was observed 6 years after the introduction of ACTs. Moreover, the 50% inhibitory concentration (IC_50_) values for LM were significantly higher in *nfs*-K65 wild-type field isolates than in the mutant 65Q isolates (25).

Mutations in the dihydrofolate reductase gene (*dhfr* 51I, 59R, and 108N triple mutant) combined with mutations in the dihydropteroate synthetase gene (*dhps* 437G and 540E double mutant) are associated with clinical and parasitological SP treatment failure in East Africa (26–29). Although SP is the drug of choice for intermittent preventive treatment in pregnant women (IPTp) (30), regional differences in drug resistance critically impact the success of this intervention (31). An additional *dhps* mutation (A581G) has been associated with reduced IPTp efficacy when the prevalence of sextuple-mutant (*dhfr* 51I/59R/108N and *dhps* 437G/540E/581G) parasites exceeds 37% (32). Also, although its impact has yet to be elucidated, a novel *dhps* I431V allele has been detected in West Africa, Cameroon, and Nigeria (33, 34).

These studies highlight the need for continued surveillance of known drug resistance markers and novel markers to maintain the gains made in using ACTs and IPTp in controlling malaria. *crt*, *mdr1,* and *dhfr* have well-described selection patterns in response to the withdrawal of CQ and SP. Based on previous findings, there was evidence of selection toward a predominance of wild-type alleles in *crt* and *mdr1* and toward the fixation of the mutant *dhfr* alleles, over a 20-year period of changing antimalarial policy (35). Therefore, the aim of this study was to use the introduction of ACTs as the pivotal point to test for evidence of selection in novel markers (*ap2-mu*, *falcipain-2a*, *k13*, *nfs,* and *ubp-1*) whose impacts are not yet fully understood.

## RESULTS

### Genetic markers associated with artemisinin resistance.

The *k13* codon K189T was the only polymorphism maintained at frequencies of >10%, while the rest of the observed alleles were rare, including codon A578S, with frequencies barely reaching 2%. Other than K189T, the only other polymorphism observed across all time points is the asparagine (Asn) repeat at codon 137. This repeat region included insertions of between one and four asparagine residues, though at low frequencies, <3%, compared to SE Asia (>60%) (36, 37). Many of the polymorphic codons occurred in the N-terminal region compared to the C-terminal region, and from 1995/1996 to 2015/2016, the *K_a_*/*K_s_* ratio for the whole *k13* gene ranged from 2.25 to 5. Conversely, the *K_a_*/*K_s_* ratio for the N-terminal region ranged from 2 to 9, while for the C-terminal region, the *K_a_*/*K_s_* ratio was 1 throughout the same time period, except for 2012/2013, when there were no polymorphisms in the C-terminal region. The observations in the N-terminal region are comparable to those from other African studies, whereas fewer mutations were identified in SE Asian parasites (16). Comparisons with the MalariaGEN data set (16) and other African studies (38, 39), revealed 9 loci that were unique to the Kilifi population and primarily occurred at only one time point over the 24-year study period. The codon K189T had frequencies similar to those of parasites examined in East Africa (13%) and West Africa (50%); however, the frequencies were much lower in SE Asia (<0.005%). Additionally, none of the *k13* mutations associated with resistance in SE Asia were identified (Table 1). Of the 13 haplotypes, the 3D7 haplotype (PK[N6]MAISKLQ) was dominant over the entire sampling period, with frequencies of >70%. The second dominant haplotype (PK[N6]MAISTLQ) showed stable frequencies from 1995/1996 to 2005/2006 (8.8% to 9.8%); however, it roughly doubled to 19.6% in 2012/2013 and then dropped back to 8.8% in 2015/2016 (see Table S2 in the supplemental material).

**TABLE 1:** *k13* SNP frequencies^*a*^

Codon	Nucleotide	Codon (nucleotide) for:		Frequency (% [no.]) during yr:				
		Wild type	Mutant	1995/1996	1998/1999	2005/2006	2012/2013	2015/2016
38	112	S (A)	C (T)	0 (132)	0 (117)	0 (133)	0 (114)	0.7 (135)
96	287	P (C)	Q (A)	0 (132)	0.7 (126)	0 (133)	0 (114)	0 (137)
108	322	K (A)	E (G)	0 (95)	0.7 (126)	0.7 (133)	0 (114)	0 (137)
119^*b*^	355	L (T)	L (C)	0.7 (132)	0 (126)	0.7 (133)	0 (114)	0 (136)
126^*b*^	377	T (C)	N (A)	0.7 (132)	0.7 (126)	0 (133)	0 (114)	0 (136)
134	401	I (T)	S (G)	0 (126)	0.7 (126)	0 (133)	0 (114)	0 (135)
136	406	H (C)	N (A)	0.7 (126)	0 (126)	0.7 (133)	1 (115)	0 (135)
137^*b*^^,^^*c*^	409	Nx6 (6xAAT)	Nx7 (7xAAT)	2 (124)	1 (126)	3 (133)	3 (115)	0.7 (134)
			Nx8 (8xAAT)	3 (124)	1 (126)	0 (133)	1 (115)	1 (134)
			Nx9 (9xTAA)	0 (124)	0 (126)	0 (133)	1 (115)	0 (134)
			Nx10 (10xTAA)	0 (124)	0 (126)	0 (133)	1 (115)	0 (134)
148	443	I (T)	T (C)	0 (122)	0 (104)	0.7 (133)	0 (114)	0 (133)
149^*b*^	445	T (A)	S (T)	0 (122)	0.7 (127)	0 (133)	0 (114)	1 (133)
157^*b*^	469	M (A)	V (G)	0 (122)	0.7 (127)	0 (133)	0 (114)	0 (132)
174	520	A (G)	S (T)	0 (95)	0 (125)	0 (133)	0.8 (115)	0 (83)
178^*b*^	532	I (A)	L (T)	1 (86)	0 (126)	0 (128)	0 (108)	0 (68)
182^*b*^	544	S (T)	T (A)	0 (95)	3 (125)	1 (132)	0 (117)	0 (87)
189^*b*^	566	K (A)	T (C)	8 (82)	15 (126)	10.6 (132)	15 (115)	13 (71)
	567	K (A)	T (T)	0 (79)	0 (124)	0.7 (132)	0.8 (115)	0 (71)
192^*c*^	574	T (A)	A (G)	0 (72)	0 (125)	0.7 (131)	0 (104)	0 (74)
258^*b*^	772	L (T)	M (A)	1 (91)	1 (100)	0 (105)	0 (79)	0.8 (116)
271^*b*^	813	Q (G)	H (T)	0 (93)	0 (69)	0.9 (107)	0 (81)	0 (121)
354	1060	I (A)	V (G)	0.7 (132)	0 (136)	0 (126)	0 (103)	0 (138)
417^*b*^	1251	P (C)	P (T)	0.7 (135)	0 (140)	0.7 (126)	0 (105)	0 (129)
469^*b*^	1407	C (C)	C (T)	0 (138)	0 (139)	2 (126)	0 (102)	0 (139)
487	1461	V (A)	V (T)	0 (139)	0.7 (127)	0 (130)	0 (105)	0 (142)
578^*b*^^,^^*c*^	1732	A (G)	S (T)	1 (137)	0.7 (127)	0.7 (127)	0 (117)	0.7 (142)
	1733	A (C)	V (T)	0.7 (137)	0 (131)	0 (127)	0 (117)	0 (142)
589^*b*^	1767	V (C)	V (T)	0 (136)	0 (123)	0 (127)	0 (117)	0.7 (139)

a The numbers of samples successfully genotyped per time point include 148 in 1995/1996, 146 in 1998/1999, 146 in 2005/2006, 132 in 2012/2013, and 148 in 2015/2016. No sequences with mixed bases were identified. Frequency data are presented as the percentage of sequences that carried a mutation out of the total number of sequences that had data for that locus (*n*). Polymorphisms in codons 30 to 417 fall in the N-terminal region, while those from 469 to 589 fall in the C-terminal region. SNPs not marked as described in footnotes b and c appear to be unique to the Kilifi parasite population. In gray are frequencies of zero.

b N-terminal SNPs that have been identified in parasites from Africa.

c N-terminal SNPs that have been identified in parasites from SE Asia.

### Other putative genetic markers associated with artemisinin resistance.

There were no mutations identified in *arps10*, *crt,* and *fd*. However, a high-frequency (>10%) single-nucleotide polymorphism (SNP) (I492V) was identified in *mdr2* in the 1995/1996 and 2015/2016 time points, and hence, the three middle time points were included. Consequently, two additional polymorphisms were identified (I495V, 0.9% in 1998/1999 and V506I, 0.7% in 2005/2006), with the I492V polymorphism maintaining high frequencies (between 13% and 30%; Table S3).

In *ap2-mu*, no significant temporal trends were observed, and only two polymorphisms achieved frequencies of >10% across time, I100I and [7N]227[6N/8N/9N/10N]. Of note, the prevalence of S160N mutation was similar from 1995/1996 to 2015/2016, except for a 2-fold increase from 9% in 1998/1999 to 24% in 2012/2013 that later decreased to 16% in 2015/2016. Over the same period, the E163E allele showed similar increases and decreases in frequency from 8.51% in 2005/2006 to 16.36% in 2012/2013 and down to 4.55% in 2015/2016) (Table S4). A total of 38 haplotypes were assembled, with the 3D7 haplotype TRSKT[N7][Kx1]AG[N5][N4]AFI dominating across time (>39%, Table S9).

*falcipain-2a* was found to be the most polymorphic gene; however, the S69Stop polymorphism was not identified (Table S5). Of the 71 haplotypes assembled, the 3D7 haplotype was not observed (Table S10). The only significant temporal trend found was that of codon S59F (*χ*^2^ = 5, *P* = 0.02), with the 59F allele dropping from 13% in 1995 and 1996 to 5% in 2015/2016. From 2005/2006 and 2012/2013 through to 2015/2016, the following alleles showed similar 2-fold increases and decreases in frequency: N4H, A8I, P9P, H10N and E11E (17% to 8% and to 15%, respectively).

In *ubp-1*, only two polymorphisms were found to exceed 10% frequency across time (range, 10 to 18%), the KYD repeat at codon 1520 and the KYE at codon 1526 (Table S6). The E1528D polymorphism observed in previous studies from Kenya was not identified. Additionally, from 2005/2006, 2012/2013, to 2015/2016, the following alleles showed 2-fold increases and decreases in frequency: the KNE repeat at codon 1514 (5.5% to 11.6% and to 5.41%, respectively) and N1518N (5.6% to 11.7% and to 5.5%, respectively). Of the 23 haplotypes, the 3D7 haplotype dominated throughout the sampling period, and no significant temporal trends were observed (Table S11).

### Genetic markers associated with CQ, SP, and lumefantrine resistance.

**FIG 1:**
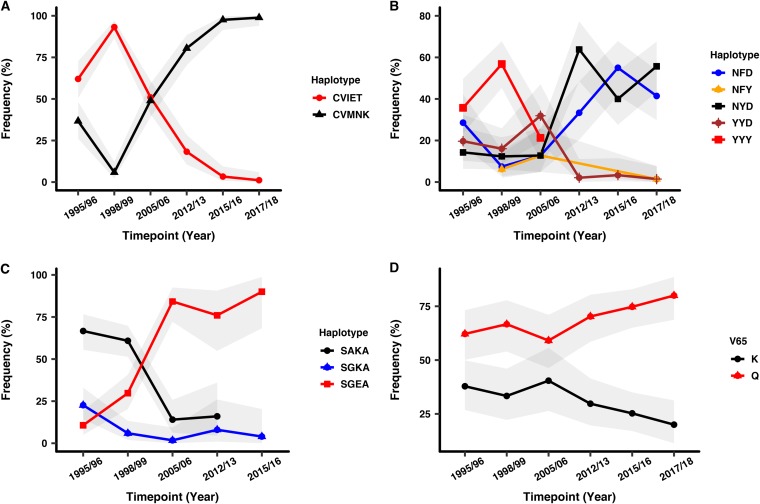
*crt*, *mdr1*, and *dhps* haplotypes and *nfs* codon K65Q frequencies over time. (A) The *crt*-sensitive haplotype (CVMNK) decreased from 1995/1996 to 1998/1999 and increased onwards to almost fixation in 2017/2018, while the *crt*-resistant haplotype (CVIET) followed an opposite pattern. (B) The 3D7 *mdr1* haplotype NYD had was the least prevalent in comparison to the mutant haplotypes NFD, YYD, and YYY pre-ACT introduction. The triple-mutant YYY was undetectable after 2005/2006, while the 3D7 NYD and mutant NFD haplotypes started to increase in the population after 2005/2006. The mutant YYD and NFY haplotypes decreased to almost zero in 2017/2018. (C) The SP-sensitive haplotype (SAKA) was on a decline from 1995/1996 and was undetectable in the population after 2012/2013. The SP-resistant double-mutant haplotype SGEA was on the increase from 1995/1996 and reached >80% frequency in 2015/2016. The single-mutant haplotype SGKA was the least prevalent throughout the sampling period. (D) The two *nfs* K65Q alleles appear to have stable frequencies from 1995/1996 to 2005/2006, but the frequency of K65 starts to drop after 2005/2006, while that of 65K starts to increase after 2005/2006. In gray are the 95% confidence intervals.

Three polymorphic codons (M74I, N75E, and K76T) were identified in the *crt* gene in 2015/2016 and 2017/2018. Mutant alleles at codons 74I, 75E, and 76T dominated during the pre-ACT period, and there was a distinct shift to the wild-type alleles (M74, N75, and K76) in the post-ACT period, almost reaching fixation (99%) (*χ*^2^ = 181, *P* < 0.001; Table 2). There was a significant decline in the frequency of the CQ-resistant (CQR) haplotype (CVIET) over time, reaching 1% in 2017 to 2018 and a sharp increase in the frequency of the CQ-sensitive (CQS) haplotype (CVMNK) from 7% in 1998/1999 to 99% in 2017/2018 (χ^2^ = 181, *P* < 0.001; Fig. 1A and Table S12).

**TABLE 2:** *crt*, *mdr1*, and *dhps* SNP frequencies^*a*^

Gene	Codon	Nucleotide	Codon (nucleotide) for:		Frequency (% [no.]) during yr:					
			Wild type	Mutant	1995/1996	1998/1999	2005/2006	2012/2013	2015/2016	2017/2018
*crt*	74	222	M (G)	I (T)	62.03 (80)	93.2 (103)	50.98 (102)	18.29 (82)	3.16 (94)	1.1 (92)
	75	223	N (A)	E (G)	62.03 (80)	93.2 (103)	50.98 (102)	18.29 (82)	3.16 (94)	1.1 (92)
	75	225	N (T)	E (A)	62.03 (80)	93.2 (103)	50.98 (102)	18.29 (82)	3.16 (94)	1.1 (92)
	76	227	K (A)	T (C)	62.03 (80)	93.2 (103)	50.98 (102)	18.29 (82)	3.19 (94)	1.1 (92)
*mdr1*	86	256	N (A)	Y (T)	57.14 (57)	72.84 (81)	57.45 (47)	2.08 (48)	2.8 (107)	1.22 (82)
	102	306	G (T)	G (C)	ND	ND	ND	ND	1.9 (105)	7.41 (81)
	182	546	G (T)	G (G)	ND	ND	ND	ND	2.56 (78)	0 (89)
	184	551	Y (A)	F (T)	30.56 (57)	13.58 (81)	29.79 (47)	33.33 (48)	54.43 (79)	42.5 (80)
	1246	3736	D (G)	Y (T)	37.5 (57)	64.2 (81)	38.3 (47)	0 (48)	2.8 (107)	1.14 (88)
*dhps*	436	1306	S (T)	A (G)	0 (85)	2.42 (124)	0 (80)	0 (21)	0 (24)	NA
	437	1310	A (C)	G (G)	32.94 (85)	37.9 (124)	86.49 (74)	78.57 (28)	0 (26)	NA
	540	1618	K (A)	E (G)	10.42 (196)	28.12 (128)	83.17 (101)	68.29 (41)	91.3 (45)	NA
	581	1742	A (C)	G (G)	0 (93)	0 (120)	0 (89)	0 (54)	3.03 (32)	NA

a The numbers of samples successfully genotyped per time point include *crt*, 103 in 2015/2016 and 91 in 2017/2018 and no sequences with mixed bases identified; *mdr1*, 130 in 2015/2016 and 88 in 2017/2018 and no sequences with mixed bases identified; *dhps*, 99 in 1995/1996, 137 in 1998/1999, 130 in 2005/2006, 80 in 2012/2013, and 72 in 2015/2016, with two sequences having mixed bases in 1998/1999. Frequency data are presented as the percentage of sequences that carried a mutation out of the total number of sequences that had data for that locus (*n*). For *dhps* 2017/2018 and *crt* (codons 102 and 182 in 1995/1996, 1998/1999, and 2005/2006, respectively), no data (NA) was available because they were not genotyped. ND, not determined. In gray are frequencies of zero.

Five polymorphic codons were identified in *mdr1* in 2015/2016 and 2017/2018, as follows: N86Y, G102G, G182G, F184Y, and D1246Y. *mdr1* codons 86Y and 1246Y also showed a distinct shift from mutant alleles in the pre-ACT period (∼40 to 70%) to wild-type alleles N86 and D1246, nearly approaching fixation (99%) (*χ*^2^ = 103, *P* < 0.001 and *χ*^2^ = 85, *P* < 0.001, respectively) post-ACT introduction. In contrast, the mutant 184F allele increased in frequency during the post-ACT period (33 to 54%, *χ*^2^ = 15.8, *P* < 0.001; Table 2). There was a notable change in haplotype frequencies between the 3D7 haplotype (NYD) and mutant NFD haplotype. NYD increased sharply to 64% in 2012/2013, decreased to 40% in 2015/2016, and later rose to 55% in 2017/2018 to become the dominant haplotype. The mutant haplotype NFD followed an opposite pattern, rising to 55% in 2015/2016 and later decreasing to 41% in 2017/2018. The triple-mutant haplotype YYY was no longer detected in the population post-ACT (Fig. 1B and Table S12), and there was no significant temporal trend observed for the haplotype frequencies. The *mdr1*-G102G and G182G identified in 2015/2016 and 2017/2018 at low frequencies (1 to 7%) were not genotyped in an earlier study (35); hence, we could not describe their associations with changing antimalarial drug policy.

S436A, A437G, K540E, and A581G *dhps* polymorphic codons were identified across all time points, and the I431V mutation was not identified. The mutant (437G and 540E) alleles dominated in the post-ACT period (68 to 91%; *χ*^2^ = 82.7, *P* < 0.001 and *χ*^2^ = 153, *P* < 0.001, respectively). The SP-sensitive haplotype (SAKA) decreased in frequency over time and was no longer detectable in 2015/2016 (Table 2), while the single-mutant haplotype (SGKA) decreased gradually to 9% in 2015/2016 and the double-mutant haplotype (SGEA) rose in frequency from 10% to 85% in 2015/2016 (Fig. 1C and Table S12). There was a significant temporal trend between the two dominant haplotypes, SAKA and SGEA (*χ*^2^ = 91, *P* < 0.001).

In *nfs*, only codon K65Q was found to have a significant, albeit marginal, trend pre- and post-ACT introduction (*χ*^2^ = 4.4, *P* = 0.04). It was also in high linkage disequilibrium with codons S62N and E67G. The 2005/2006 period appears to be the point at which the mutant allele 65Q and wild-type 65K diverge in frequency in opposite directions, with the wild-type 65K decreasing and the mutant allele 65Q increasing in frequency (Fig. 1D and Table S7). The 3D7 haplotype was the seventh most dominant of all the 24 haplotypes, and there were no significant temporal trends in haplotype frequencies (Table S13). Additionally, we examined for spatial variation for *crt*, *mdr1,* and *nfs* and did not see any variation in alleles according to geographical area (Table S15).

### serine-tRNA ligase, putative gene.

Serine-tRNA ligase, a marker not associated with resistance or drug selection, had only one polymorphic codon observed across all time points (L84V), with frequencies ranging between 2 and 5%, while the rest were rare (<5%) (Table S8). A total of 15 haplotypes were observed, and only the 3D7 haplotype occurred across all time points and with frequencies of >80%, with the rest of the haplotypes being rare (<5%) (Table S14).

## DISCUSSION

The stable frequencies of the common *k13* A578S African mutation and K189T (16, 40, 41) suggest that these mutations are not under selective pressure from artemisinin. Notably, a recent study confirmed that the A578S mutation does not confer resistance to artemisinin (41). The codon 137 Asn repeats, found at lower frequencies than in SE Asia, have been associated with day 3 positivity (36) and cooccur with *k13* propeller mutants (37). However, it is not known how these polymorphisms modulate artemisinin resistance, and further studies are needed to investigate this. Similar to parasites from Africa, the N-terminal region of *k13* had higher *K_a_*/*K_s_* ratios than did the C-terminal region, contrary to *k13* data from SE Asia (16), implying that parasites from Africa acquire more changes in the N-terminal region than the C-terminal region. Moreover, as has been observed in parasites from Africa (16), there was no artemisinin resistance-predisposing mutations, and the *mdr2* I492V mutation showed no evidence of selection over time. This mutation (I492V) has also been identified at 100% frequency (*n* = 38) in Suriname (42).

The withdrawal of CQ (43) has resulted in the rapid decline in CQR alleles *crt* 76T and *mdr1* 86Y and *mdr1* 1246Y in Kilifi. Nationally, a decline in CQR resistant alleles has previously been observed on the South Coast of Kenya, with the *crt* 76T and *mdr1* 86Y alleles decreasing from 88% to 63% and 75% to 54% from 1998 to 2008, respectively (44). In western Kenya, where malaria transmission is holoendemic, the *crt* 76T, *mdr1* 86Y, and *mdr1* 1246Y alleles dropped from 86% to 2%, 92% to 1%, and 67% to 6% between the years 2003 and 2014, respectively (45). In the same region, CQ median IC_50_ values decreased significantly, from a median of 92 to 22 from 2008 to 2011 (*P < *0.001) (46). Therefore, a national rerollout of CQ in the next few years is a possibility. However, it is also likely that resistant parasites may remain in the population below detectable levels, and reemergence from these parasites or the reintroduction from surrounding areas could be rapid. Regarding *mdr1*, the disappearance of the triple-mutant *mdr1*-YYY is also likely to be the result of CQ withdrawal. Likewise, the oscillation of the *mdr1*-NFD with the NYD (wild type) at a high frequency post-ACT introduction suggests that the NYD haplotype is likely to have risen in frequency due to the absence of CQ, restoring the wild-type parasite population, while the increase in the NFD haplotype frequency may be attributable to artemether-lumefantrine (AL) pressure (19).

The high frequency of SP resistance markers in Kenya (35) may be attributable to the continued distribution of SP for malaria case management in the private sector (47). Though SP maintains its utility in IPTp, the loss of IPTp efficacy has been noted when the prevalence of the sextuple-mutant (*dhfr* 51I/59R/108N and *dhps* 437G/540E/581G) parasites exceeds 37% (32), as seen in some sub-Saharan African settings. Consequently, the *dhps* 581G mutation needs to be monitored, given that it first occurs in our population in 2015/2016 at 3%. The *dhps* I431V allele was not detected, and its distribution could be restricted to West Africa (33, 34).

We noted a decline in the *nfs* K65 wild-type allele in the Kilifi population since the introduction of AL in 2004, contrary to recent findings from The Gambia in West Africa (25), and it appears that there is an opposite trend of the K65 allele in this East African population. The Gambian parasites have shown increasing tolerance to lumefantrine (LM) in a study conducted between 2013 and 2015 (48); however, drug trials with AL in western Africa, including The Gambia, show that AL is still highly efficacious (49). Perhaps these discordant observations are due to differences in allele frequencies of other loci, such as *pfmdr1,* between East and West Africa (50), or differences in drug policy, such as the extensive use of amodiaquine (AQ) in West Africa compared to East Africa (51). AQ shows activity against CQR parasites, like in West Africa, where the prevalence of CQR parasites is still high (52). However, in East Africa, there are reports of AQ resistance (53, 54) and AQ-resistant parasites are sensitive to LM (19). Thus, this geographical difference in drug use, the inverse resistance profile between AQ and LM, and the predominance of CQS parasites in East Africa suggest that AQ may drive LM resistance in West Africa.

The distinct change in frequency of resistance-associated alleles observed with *crt*, *mdr1*, *dhps,* and *nfs* were not seen in the other genes we evaluated, *ap2-mu*, *falcipain-2a*, and *ubp-1*. Notably, *ap2-mu* 160N showed no evidence of selection and has not been observed in parasites in SE Asia, where artemisinin resistance is common (16). Therefore, it appears to be restricted to Africa, as it has also been observed in Ghana (55). Recent evidence points to a different mutation, I592T (not detected in this population), that showed increased ring-stage survival following a dihydroartemisinin pulse (56). Further work is required to evaluate the potential role of *ap2-mu* mutations on the ACT response.

*falcipain-2a* was the most polymorphic gene in this study, and this can be attributed to drug pressure, as it is associated with the parasite’s digestive vacuole, the target of many antimalarials (57). Nonetheless, The S69Stop mutation that results is artemisinin resistance *in vitro* (15) was not identified in our population. However, we found a significant temporal increase in the S59 allele post-ACT introduction, potentially due to AL pressure. It remains to be seen what the role of *falcipain-2a* is in modulating artemisinin resistance.

We did not identify the *ubp-1* 1528D mutation, contrary to a study by Henriques et al. (21), which showed that its frequency increased post-ACT treatment, although it has been found at a prevalence of 7.4% in Ghana (55). A different *ubp-1* SNP (R3138H), outside the region we genotyped, has also been associated with artemisinin resistance on the Thai-Myanmar border (Cerqueira et al. [58]). Like *ap2-mu* and *falcipain-2a*, this calls for further work to understand the role of *ubp-1* as a marker of ACT resistance.

We observed 2-fold increases or decreases in the frequencies of alleles and haplotypes of several genes, followed by a shift back to the original frequency in the durations spanning 2012/2013, 2015/2016, and through to 2017/2018. This could be due to the abrupt change in drug policy from SP to ACTs and hence the initial selection of long haplotypes followed by recombination breaking down the haplotype structure as the parasite adapts to changes in drug pressure. The lack of spatial variation in allele frequency is consistent with previous reports at this geographical scale (59); however, we had limited power to do a more detailed analysis beyond north and south Kilifi.

Following the introduction of ACTs in 2004, there has been a rapid increase in the CQ-sensitive population to near fixation, and this reignites the debate on the use of CQ for malaria treatment, such as in combination therapy. On the other hand, there is still a need for careful monitoring of the *dhps* A581G locus, since SP has proved useful in IPTp, significantly reducing morbidity in pregnant women (32). The decline in the novel marker (*nfs*), which potentially confers resistance to LM, contrary to the observations made in The Gambia, calls for additional studies to determine its role as a potential drug target. The artemisinin resistance-conferring SE Asian mutations in *k13*, such as C580Y, have not been identified in Kilifi, and many of the SNPs occurred in the N-terminal region of the gene with no evidence of drug selection. Consequently, due to a lack of the validated molecular markers of artemisinin and lumefantrine resistance, there appears to be no problem of resistance in the population; however, continued surveillance remains a requirement.

## MATERIALS AND METHODS

### Study design.

**FIG 2:**
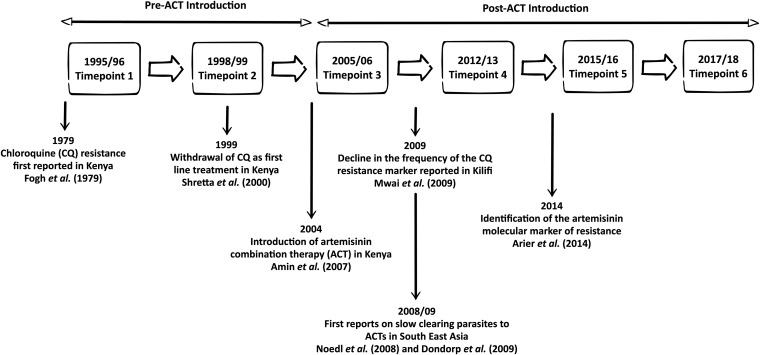
Schematic showing the time points from which parasite populations were genotyped. Also indicated are historical highlights of antimalarial drug resistance in Kilifi versus South East Asia. Cited are studies by Noedl et al. (7), Dondorp et al. (8), Ariey et al. (15), Shretta et al. (43), Mwai et al. (64), Amin et al. (65), and Fogh et al. (66).

We used parasite DNA extracted from frozen blood obtained in 1995/1996, 1999/2000, 2006/2007, and 2012/2013 from a previous study (35) and from additional samples from 2015/2016 and 2017/2018 from this new study. One hundred fifty samples were collected for each time point, except for 2017/2018, in which 109 samples were used. Blood samples were obtained from patients presenting to Kiliﬁ County Hospital with malaria (1 month to 15 years of age). The samples span 24 years of changing drug policy (Fig. 2). Ethical approval for this study was obtained from the ethics review committee of the Kenya Medical Research Institute under protocol number SERU 3149.

### Sample preparation, PCR, and capillary sequencing.

P. falciparum genomic DNA was extracted from frozen blood using the Qiagen DNA blood minikit (Qiagen, UK), as per the manufacturer’s instructions. Amplicons were generated from the following genes using primers from previous studies and primers designed in this study (Table S1): *crt* (PF3D7_0709000), *mdr1* (PF3D7_0523000), *dhps* (PF3D7_0810800), *nfs* (PF3D7_0727200), *k13* (PF3D7_1347700), *ap2mu* (PF3D7_1218300), *falcipain-2a* (PF3D7_1347700), *ubp-1* (PF3D7_0104300), and serine-tRNA ligase, putative gene (PF3D7_0717700). Additionally, four artemisinin resistance-predisposing mutations in *arps10* (PF3D7_1460900), *crt* (PF3D7_0709000), *fd* (PF3D7_1318100), and *mdr2* (PF3D7_1447900) were genotyped. Given that these mutations in these genes have been identified at low frequencies (<1%) in Africa (16), genotyping was first done for the 1995/1996 and 2015/2016 time points, and then the remaining time points were included if SNPs with >10% frequency were identified. We used the Expand high-fidelity PCR system, 0.5 μl of template DNA, and primers and PCR conditions indicated in Table S1. The final reaction volume was 10 μl, and the PCR amplification products were visualized on 1% agarose gels stained with RedSafe nucleic acid staining solution (iNtRON Biotechnology DR). PCR-negative samples were taken through a second and final round of PCR with 0.75 μl of template DNA. Positive PCR products were purified using ExoSAP-IT (Thermo Fisher Scientific) and directly sequenced using the PCR primers, additional internal primers (Table S1), and BigDye Terminator v3.1 cycle sequencing kit v3.1 (Applied Biosystems, UK). Capillary sequencing was done at the International Livestock Research Institute (ILRI, Kenya) using an ABI 3730xl capillary sequencer (Applied Biosystems).

### Sequence analysis.

Sequence assembly was performed in CLC Main Workbench v7.9.1 (Qiagen, UK), and SNPs were identified and called based on the respective 3D7 reference sequences. Nucleotide positions which displayed a peak within a peak in the sequence chromatograms were noted as “mixed.” Consensus sequences were extracted from the sequence assemblies using CLC Genomics Workbench v9.5.3 and used to construct multiple-sequence alignments in Clustal Omega v1.2.1 (60). SNP frequencies were calculated per gene per time point, and singletons were confirmed by an additional round of PCR and sequencing.

### Statistical analysis.

Nucleotide sequences were translated into amino acid sequences in AliView v1.26 (61). Haplotypes were then generated based on the amino acid residues from all the polymorphic codons that cut across all sequences and time points after excluding sequences with mixed bases. Data for *crt* and *mdr1* from the 1995 to 2013 time points were obtained from a previously published study (35). The difference in the prevalences of alleles and haplotypes in pre-ACT and post-ACT periods was evaluated using a chi-square test. For this analysis, 2005/2006 was used as the point to divide the data into the pre- and post-ACT periods, since we do not expect complete ACT coverage following its formal introduction in 2004; hence, data for 2005/2006 were excluded from this part of the analysis. Additionally, it was the crossover point for the shift in high frequency from the *crt* 76T to *crt* K76 allele. The chi-square test for alleles included data for all SNPs with >10% frequencies at any time point, while for haplotypes, we included only the sequences that had data across all loci and with frequencies of >10% at any time point. Additionally, the chi-square test for haplotypes was conducted only for the two dominant haplotypes. All plots were generated using ggplot2 v3.1.1 (62) and ggpubr v0.2 packages (63) in R v3.6.0 (R Core Team, 2014). The *K_a_*/*K_s_* ratio was calculated for *k13* by dividing the number of nonsynonymous substitutions per nonsynonymous site (*K_a_*) by the number of synonymous substitutions per synonymous site over time.

### Data availability.

The nucleotide sequence data reported in this paper are available in the GenBank database under the accession numbers MN373285 to MN373771 (*ap2-mu*), MN373772 to MN374234 (*dhps*), MN374235 to MN374817 (*falcipain-2a*), MN374818 to MN375065 (*fd*), MN375066 to MN375759 (*k13*), MN375760 to MN376049 (*mdr1*), MN376050 to MN376651 (*mdr2*), MN376652 to MN377106 (*nfs*), MN377107 to MN377726 (serine tRNA ligase gene), and MN377727 to MN378298 (*ubp1*). However, sequences for *arps10* and *crt* could not be deposited, as GenBank does not accept sequences under 200 bp.

## Supplementary Material

Supplemental file 1
