# Supplementary material for: No Evidence of Plasmodium falciparum k13 Artemisinin Resistance-Conferring Mutations over a 24-Year Analysis in Coastal Kenya but a Near Complete Reversion to Chloroquine-Sensitive Parasites
Source: Antimicrob Agents Chemother. 2019 Nov 21;63(12):e01067-19. doi: 10.1128/AAC.01067-19 (PMC6879256; doi:10.1128/AAC.01067-19)
Supplement: Supplemental file 1 [file AAC.01067-19-s0001.docx]

## Supplementary Tables

**Supplementary Table 1.** List of primers for PCR and sequencing

| **Gene** | **Primer ID** | **Primer Sequence in ‘5-3’ Orientation** | **PCR Annealing Temperature** | **Reference** |
| --- | --- | --- | --- | --- |
| *ap2-mu* (full length) | ap2mu-1F* | GTT AAC ACG ATT AGC GTC ATT TG | 54°C for 2 min | Henriques et al., (2014) |
|  | ap2mu-2R | GTC CTA TTA TGT ATA TGT GGA TC |  |  |
|  | ap2mu-3F | GAT ATC CAC AAA CAT TAG AAG TG |  |  |
|  | ap2mu-4R | CCA TCT GGT GGT GTG AAG G |  |  |
|  | ap2mu-5F | GCA TAT TTC ATC ATT GTG TTA CC |  |  |
|  | ap2mu-6R* | ACA CCC GAT TGA ACT ATT TAT AC |  |  |
| *crt* (codon 72-76) | F* | GGT GGA GGT TCT TGTCTT GG | 52°C for 30 sec | Okombo et al., (2014) |
|  | R* | ATA AAG TTG TGA GTT TCG GAT G |  |  |
| *dhps* (codons A437G and K540E and A581G) | F* | CCT AAA CGT GCT GTT CAA AGA ATG | 58°C for 30 sec | Designed in this study |
|  | R* | CAT CCA ATT GTG TGA TTT GTC CAC |  |  |
| *falcipain-2a* (full length) | fp2F* | TGT AGC AAG AAC GTT TTG TGT AAA T | 56°C for 2 min | Conrad et al., (2014) |
|  | fp2NF | TGT GTA AAT TAA AGA TAA AAG TGC AAA |  |  |
|  | fp2R* | GGT AAA GGA AAA ATT AGT AAG GAT GC |  |  |
|  | fp2intR | GCA TAT TGT GAT TCT ACG GAA CC |  |  |
|  | fp2intF | AAA AAG CCC TAA TGG CAA GAA |  |  |
|  | fp2NR | GGT CCC TTT TTA AAA TAC TAT TGA CA |  |  |
| *k13* (full length) | *k13*-F* | ATG GAA GGA GAA AAA GTA AAA AC | 56°C for 30 sec | Designed in this study |
|  | *k13*-F2* | GCA GCA AAT CTT ATA AAT GAT G |  |  |
|  | *k13*-F3 | GAA GCC TTG TTG AAA GAA GC |  |  |
|  | *k13*-F4 | CAT AGG AAA CGA TTT GAT G |  |  |
|  | *k13*-F1 | CAA AAG CAA ATA GTA TCT CG |  |  |
|  | *k13*-R | CTC TTT TTT GTT GGT ATT CAT AAT TG |  |  |
|  | *k13*-R1* | CAC TAG CAT CAC TTA ATT CCG |  |  |
|  | *k13*-R2 | CAC ATA CGC CAG CAT TG |  |  |
|  | *k13*-R3* | CGG AGT GAC CAA ATC TGG |  |  |
|  | *k13*-R4 | CAT AGG AAA CGA TTT GAT G |  |  |
| k13 background - *arps10* (codon V127M) | arps-F* | CAC AAT ATT ATG TTT CAT TTT AG | 55°C for 30 sec | Designed in this study |
|  | arps-R* | GTA TAA TTT ATT CTG CTT ACA TTC |  |  |
| k13 background - *crt* (codon I356T) | *crt*-F*  *crt*-R* | GAT TAT CGA CAA ATT TTC TAC  CTT TTT AAT TCT TAC GGC TAA G | 55°C for 30 sec |  |
| k13 background - *fd* (codon *D193Y)* | fd-F* | GAT GCT AGT GAA AGA CAG AAT G | 55°C for 30 sec |  |
|  | fd-R* | CAC ATA TTT TTG ATT GAG GAC |  |  |
| k13 backgroun*d* - *mdr2* (codon T484T) | *mdr2*r-F* | GAG GTT TGT GGT GTA TTA TTT TC | 55°C for 30 sec |  |
|  | *mdr2*r-R* | CAT TAT CCA TCT CAT TTG CTT TTG |  |  |
| *mdr1* | F1* | ATG GGT AAA GAG CAG AAA G | 51°C for 30 sec | Okombo et al., (2014) |
|  | R2* | TCC CAT TAA AGC CTC TTC TA |  |  |
|  | F2* | ATT GAT GTA AGA GAT GAT GGT |  |  |
|  | R1* | TAT TCC ATC TTG TGC TGA TAA |  |  |
| *nfs* | F1* | CTT CAA TTT TGT AAT GAA ATT TCT TC | 51°C for 30 sec | Designed in this study |
|  | R1* | CAT ATT ATG TCC ATA TAA ACT TTG GA |  |  |
|  | F2 | ATG CAC GAA CAA ATC TTT TGA |  |  |
|  | R2 | AGA TTT GTT CGT GCA TCC T |  |  |
| *ubp-1* | F* | CGC CCG TAC TAT GAA GAA GAT C | 50°C for 2 min | Henriques et al., (2014) |
|  | R* | CCG TTT TAC CTG AAC TGT TCA GG |  |  |
| *serine-tRNA ligase, putative* | RNAligase_F1* | ATG GTT TTA GAT ATA AAT TTA TTT CG | 55°C for 30 sec | Designed in this study |
|  | RNAligase_F2 | GAA AAA TAG GTG GAG CTA |  |  |
|  | RNAligase_F3 | ATG ATA CAA ACA TGT GAA GA |  |  |
|  | RNAligase_R1 | TCT TGT TCA CTC CAT AAA GGG A |  |  |
|  | RNAligase_R2* | GTA ATA AGA AAC CTG CAC CTG |  |  |

All primers were used for sequencing while those marked with ***** were used for both PCR and sequencing. The taq polymerase used in this study (Expand™ High Fidelity PCR system) has two rounds of PCR including a first round that was set at 10 cycles and the second set at 25 cycles. The following conditions were used including the annealing temperatures indicated above: denaturation (94°C – 15 sec), annealing (gene specific), extension (72°C – 2 min), and final extension (72°C – 7 min).

**Supplementary Table 2.** *k13* haplotype frequencies

| Haplotype | Frequency % [n] | | | | |
| --- | --- | --- | --- | --- | --- |
|  | 1995/96 | 1998/99 | 2005/06 | 2012/13 | 2015/16 |
| PK[N6]MAISKLQ* | 76.4 [34] | 75 [56] | 82.9 [94] | 71.4 [56] | 88.2 [34] |
| PK[N6]MAISTLQ | 8.8 [34] | 12.5 [56] | 9.5 [94] | 19.6 [56] | 8.8 [34] |
| PK[N7]MAISKLQ | 5.8 [34] | 1.7 [56] | 2.1 [94] | 3.5 [56] | 2.9 [34] |
| PK[N6]MAISKMQ | 2.9 [34] | 1.7 [56] | 0 [94] | 0 [56] | 0 [34] |
| PK[N6]MALSKLQ | 2.9 [34] | 0 [56] | 0 [94] | 0 [56] | 0 [34] |
| PK[N8]MAISKLQ | 2.9 [34] | 0 [56] | 0 [94] | 3.5 [56] | 0 [34] |
| PK[N6]MAITKLQ | 0 [34] | 5.3 [56] | 2.1 [94] | 0 [56] | 0 [34] |
| PK[N6]VAISKLQ | 0 [34] | 1.7 [56] | 0 [94] | 0 [56] | 0 [34] |
| QK[N6]MAISKLQ | 0 [34] | 1.7 [56] | 0 [94] | 0 [56] | 0 [34] |
| PE[N6]MAISKLQ | 0 [34] | 0 [56] | 1.0 [94] | 0 [56] | 0 [34] |
| PK[N6]MAISNLQ | 0 [34] | 0 [56] | 1.0 [94] | 0 [56] | 0 [34] |
| PK[N6]MAISTLH | 0 [34] | 0 [56] | 1.0 [94] | 0 [56] | 0 [34] |
| PK[N6]MSISKLQ | 0 [34] | 0 [56] | 0 [94] | 1.7 [56] | 0 [34] |

The following were used to define haplotypes: 10 polymorphic sites, including codons 96, 108, 137, 157, 174, 178, 182, 189, 258 and 271. The haplotype marked with an * indicate the 3D7 haplotype. Frequency is presented as the percentage of sequences that contributed to the haplotype out of the total number of sequences [n]. In grey are zero SNP frequencies.

**Supplementary Table 3.** *mdr2* SNP frequencies

| Codon | Nucleotide | Codon [Nucleotide] | | Mutant Frequency % [n] | | | | |
| --- | --- | --- | --- | --- | --- | --- | --- | --- |
|  |  | Wildtype | Mutant | 1995/96 | 1998/99 | 2005/06 | 2012/13 | 2015/16 |
| 492 | 1474 | I [A] | V [G] | 30.5 [131] | 21 [108] | 13 [132] | 14 [116] | 23 [130] |
| 495 | 1483 | I [A] | V [G] | 0 [131] | 0.9 [108] | 0 [139] | 0 [116] | 0 [130] |
| 506 | 1516 | V [G] | I [A] | 0 [129] | 0 [107] | 0.71 [136] | 0 [115] | 0 [130] |

The number of samples successfully genotyped per timepoint include: 131 in 1995/96, 117 in 1998/99, 134 in 2005/06, 145 in 2012/13 and 130 in 2015/16. No sequences with mixed bases identified. Frequency is presented as the percentage of sequences that carried a mutation out of the total number of sequences that had data for that locus [n]. In grey are zero SNP frequencies.

**Supplementary Table 4.** *ap2-mu* SNP frequencies

| Codon | Nucleotide | Codon [Nucleotide] | | Mutant Frequency % [n] | | | | | |  |
| --- | --- | --- | --- | --- | --- | --- | --- | --- | --- | --- |
|  |  | Wildtype | Mutant | | 1995/96 | 1998/99 | 2005/06 | 2012/13 | 2015/16 | |
| 44 | 132 | E [A] | E [G] | | 0 [93] | 0 [110] | 2 [48] | 0 [43] | 0 [77] | |
| 92 | 275 | F [T] | S [C] | | 0 [87] | 0 [110] | 2 [48] | 0 [48] | 0 [81] | |
| 100 | 300 | I [A] | I [C] | | 29 [86] | 30 [106] | 37 [37] | 36 [46] | 31 [86] | |
| 127 | 381 | V [G] | V [A] | | 0 [74] | 0 [101] | 0 [21] | 2 [49] | 0 [92] | |
| 144 | 430 | T [A] | A [G] | | 0 [75] | 0 [102] | 3 [27] | 0 [51] | 0 [90] | |
| 146 | 437 | R [G] | K [A] | | 0 [74] | 1 [102] | 3 [27] | 0 [51] | 0 [91] | |
| 149 | 447 | Q [G] | Q [A] | | 1 [76] | 1 [101] | 3 [27] | 0 [52] | 2 [92] | |
| 160 | 479 | S [G] | N [A] | | 10 [75] | 11 [102] | 9 [44] | 24 [44] | 16 [109] | |
| 162 | 486 | I [T] | I [C] | | 0 [77] | 3 [103] | 0 [47] | 0 [55] | 1 [110] | |
| 163 | 489 | E [A] | E [G] | | 7 [77] | 12 [102] | 8 [47] | 16 [55] | 4 [110] | |
| 188 | 564 | R [A] | R [G] | | 1 [85] | 0 [104] | 0 [51] | 0 [57] | 0 [117] | |
| 199 | 596 | K [A] | T [C] | | 11 [86] | 6 [102] | 0 [59] | 16 [60] | 11 [114] | |
|  | 597 | K [A] | K [G] | | 0 [86] | 0 [102] | 3 [59] | 0 [60] | 0.8 [114] | |
| 200 | 598 | N [A] | Y [T] | | 1 [87] | 0 [102] | 1 [60] | 0 [60] | 0 [114] | |
| 207 | 620 | T [C] | R [G] | | 1 [89] | 0.9 [102] | 0 [59] | 0 [60] | 0 [115] | |
| 225 | 675 | I [A] | I [T] | | 0 [90] | 0 [101] | 0 [57] | 1 [63] | 0 [114] | |
| 227 | 679 | Nx7 [7xAAT) | Nx6 [6xAAT] | | 0 [81] | 0.9 [81] | 0 [57] | 0 [65] | 0 [116] | |
|  |  |  | Nx8 [8xAAT] | | 10 [81] | 13.7 [81] | 17 [57] | 12 [65] | 14 [116] | |
|  |  |  | Nx9 [9xAAT] | | 6 [81] | 5.8 [81] | 7 [57] | 1 [65] | 12 [116] | |
|  |  |  | Nx10 [10xAAT] | | 0 [81] | 0.9 [81] | 0 [57] | 0 [65] | 0.8 [116] | |
| 234 | 700 | Kx1 [AAG] | Kx2 [2xAAG] | | 2 [91] | 2 [101] | 0 [57] | 10 [65] | 1 [111] | |
| 235 | 704 | T [C] | I [T] | | 1 [89] | 0 [100] | 0 [52] | 0 [65] | 0 [112] | |
| 236 | 706 | A [G] | T [A] | | 1 [88] | 0 [100] | 1 [51] | 1 [66] | 0 [112] | |
| 254 | 760 | Y [T] | H [C] | | 1 [85] | 0 [102] | 0 [51] | 0 [65] | 0 [112] | |
| 274 | 821 | S [G] | I [T] | | 0 [83] | 0 [104] | 2 [47] | 0 [66] | 0 [109] | |
| 289 | 867 | K [A] | K [G] | | 1 [82] | 0 [99] | 0 [47] | 0 [66] | 0 [109] | |
| 315 | 943 | G [G] | S [A] | | 0 [85] | 0 [96] | 1 [52] | 0 [70] | 0 [111] | |
|  | 944 | G [G] | D [A] | | 1 [85] | 1 [96] | 0 [52] | 0 [70] | 0 [111] | |
| 320 | 958 | Nx5 [AAC(4xAAT)] | Nx6 [AAC(5xAAT)] | | 6 [81] | 2 [76] | 10 [52] | 7 [69] | 3 [105] | |
|  |  |  | Nx7 [AAC(6xAAT)] | | 0 [81] | 2 [76] | 0 [52] | 0 [69] | 0 [106] | |
| 327 | 979 | Nx4 [(3xAAT)AAC] | Nx5 [(4xAAT)AAC] | | 2 [75] | 1 [84] | 2 [50] | 0 [66] | 2 [107] | |
|  |  |  | Nx6 [(5xAAT)AAC] | | 0 [75] | 0 [84] | 0 [50] | 0 [66] | 0.9 [107] | |
| 337 | 1009 | A [G] | T [A] | | 0 [78] | 1 [81] | 0 [46] | 1 [71] | 0 [113] | |
| 341 | 1023 | S [T] | S [C] | | 0 [77] | 1 [81] | 0 [45] | 2 [69] | 0 [113] | |
| 437 | 1311 | F [C] | L [A] | | 9 [72] | 0 [89] | 4 [65] | 4 [97] | 1 [118] | |
| 445 | 1334 | N [A] | S [G] | | 0 [75] | 0 [91] | 1 [66] | 0 [97] | 0 [118] | |
| 476 | 1428 | S [T] | S [G] | | 1 [79] | 6 [99] | 2 [79] | 0.9 [101] | 1.6 [121] | |
| 478 | 1434 | V [A] | V [T] | | 2 [78] | 4 [103] | 2 [79] | 1.9 [101] | 2.4 [121] | |
| 491 | 1473 | V [T] | V [T] | | 0 [80] | 0 [96] | 0 [76] | 0 [100] | 0.8 [122] | |
| 498 | 1493 | I [T] | T [C] | | 0 [80] | 2 [95] | 0 [74] | 0 [98] | 0 [121] | |
| 517 | 1551 | F [C] | F [T] | | 0 [84] | 1 [95] | 0 [73] | 0 [94] | 0 [119] | |
| 548 | 1644 | D [C] | D [T] | | 0 [84] | 0 [86] | 0 [66] | 0 [89] | 0.9 [111] | |
| 586 | 1758 | T [C] | T [A] | | 0 [31] | 0 [64] | 0 [58] | 0 [79] | 0.9 [104] | |
| 603 | 1809 | Y [T] | Y [C] | | 0 [4] | 0 [47] | 1 [54] | 0 [72] | 0 [100] | |

The number of samples successfully genotyped per timepoint include: 130 in 1995/96, 134 in 1998/99, 123 in 2005/06, 110 in 2012/13 and 135 in 2015/16. There were a total of 13, 16, 3,3 and 13 sequences with mixed bases in 1995/96, 1998/99, 2005/06, 2012/13 and 2015/16, respectively. Frequency is presented as the percentage of sequences that carried a mutation out of the total number of sequences that had data for that locus [n]. In grey are zero SNP frequencies.

**Supplementary Table 5.** *falcipain-2a* SNP frequencies

| Codon | Nucleotide | Codon [Nucleotide] | | Mutant Frequency % [n] | | | | |
| --- | --- | --- | --- | --- | --- | --- | --- | --- |
|  |  | Wildtype | Mutant | 1995/96 | 1998/99 | 2005/06 | 2012/13 | 2015/16 |
| 4 | 10 | N [A] | H [C] | 9 [91] | 9 [113] | 19 [73] | 9 [98] | 16 [109] |
| 8 | 22 | A [G] | I [A] | 8 [93] | 7 [113] | 16 [74] | 8 [100] | 14 [110] |
|  | 23 | A [C] | I [T] | 8 [93] | 11 [113] | 17 [74] | 8 [100] | 15 [110] |
| 9 | 27 | P [C] | P [G] | 8 [94] | 7 [113] | 17 [74] | 6 [100] | 14 [110] |
| 10 | 28 | H [C] | N [A] | 8 [96] | 8 [112] | 17 [74] | 7 [99] | 14 [110] |
| 11 | 33 | E [A] | E [G] | 8 [96] | 9 [112] | 18 [74] | 7 [99] | 15 [111] |
| 15 | 45 | Q [A] | H [T] | 10 [98] | 13 [113] | 14 [78] | 13 [99] | 13 [113] |
| 21 | 63 | V [T] | V [C] | 11 [98] | 15 [113] | 18 [79] | 13 [101] | 15 [112] |
| 31 | 93 | K [G] | K [A] | 2 [99] | 6 [116] | 3 [82] | 1 [101] | 0.8 [116] |
| 43 | 129 | S [T] | S [A] | 10 [100] | 8 [116] | 5 [81] | 4 [102] | 4 [116] |
| 47 | 141 | V [T] | V [A] | 12 [101] | 11 [115] | 11 [80] | 8 [103] | 8 [115] |
| 48 | 142 | V [G] | I [A] | 0 [142] | 0 [115] | 0 [81] | 0 [103] | 0.8 [115] |
| 51 | 151 | V [G] | I [A] | 13 [104] | 13 [115] | 13 [81] | 9 [104] | 10 [118] |
| 59 | 176 | S [C] | F [T] | 12 [105] | 8 [116] | 7 [80] | 1 [104] | 5 [118] |
| 60 | 179 | R [G] | K [A] | 0 [105] | 0 [117] | 0 [79] | 0.9 [102] | 0.8 [119] |
| 82 | 244 | L [T] | L [C] | 0.9 [106] | 0.8 [117] | 0 [81] | 0.9 [103] | 0 [118] |
| 85 | 255 | S [C] | S [T] | 0 [106] | 0 [119] | 0 [81] | 0.9 [102] | 0 [118] |
| 86 | 256 | P [C] | T [A] | 0 [106] | 0 [119] | 0 [81] | 0 [103] | 0 [118] |
| 87 | 260 | N [A] | N [C] | 0 [106] | 0.8 [119] | 0 [79] | 0 [102] | 0 [118] |
| 114 | 340 | N [A] | D [G] | 0 [103] | 0 [120] | 1 [83] | 0 [101] | 0 [118] |
|  | 342 | N [C] | N [T] | 1 [103] | 0 [120] | 0 [83] | 0 [101] | 0 [118] |
| 115 | 344 | E [A] | V [T] | 2 [103] | 3 [120] | 1 [86] | 0 [101] | 0.8 [118] |
| 123 | 369 | D [C] | D [T] | 0.9 [105] | 0 [120] | 0 [86] | 0 [102] | 0 [119] |
| 126 | 377 | G [G] | D [A] | 0 [105] | 3 [122] | 0 [86] | 0 [103] | 0 [120] |
| 127 | 381 | L [T] | L [A] | 0.9 [106] | 0 [122] | 0 [86] | 0.9 [103] | 0 [120] |
| 134 | 401 | T [C] | K [A] | 0.9 [105] | 0 [123] | 0 [89] | 0.9 [106] | 0 [120] |
| 140 | 418 | I [A] | L [C] | 0.9 [106] | 0 [124] | 0.9 [105] | 0 [108] | 0 [120] |
| 143 | 427 | K [A] | E [G] | 0 [107] | 0 [125] | 0.9 [105] | 1 [108] | 0 [120] |
| 144 | 430 | D [G] | N [A] | 0 [106] | 0 [125] | 0.9 [105] | 1 [108] | 0 [120] |
| 150 | 449 | F [T] | Y [A] | 0 [106] | 0.8 [125] | 0 [105] | 0 [111] | 0 [120] |
| 167 | 500 | M [T] | T [C] | 0.9 [107] | 0.8 [122] | 0 [98] | 0 [112] | 0 [120] |
| 169 | 507 | I [T] | I [A] | 0.9 [106] | 0.8 [122] | 0 [98] | 0 [112] | 0 [120] |
| 173 | 519 | N [T] | K [A] | 1 [105] | 2 [122] | 1 [98] | 0.8 [112] | 4 [120] |
| 179 | 537 | P [A] | P [G] | 0 [106] | 2 [123] | 2 [98] | 6 [112] | 2 [118] |
| 183 | 549 | K [G] | K [A] | 0 [106] | 0 [122] | 1 [98] | 0 [114] | 0 [119] |
| 204 | 612 | N [T] | K [G] | 0 [104] | 0.8 [122] | 0 [98] | 0 [113] | 0 [122] |
| 210 | 628 | E [G] | Q [C] | 0 [104] | 0 [122] | 0 [98] | 0.8 [114] | 4 [121] |
| 212 | 636 | N [C] | N [T] | 0 [104] | 0.8 [122] | 0 [97] | 0 [114] | 0 [121] |
| 215 | 645 | A [C] | A [T] | 0 [104] | 0.8 [124] | 0 [97] | 0 [113] | 0 [121] |
| 218 | 654 | T [T] | T [A] | 0 [105] | 0 [124] | 0 [97] | 0.8 [113] | 0.8 [121] |
| 220 | 660 | H [T] | H [C] | 0 [105] | 0.8 [124] | 0 [97] | 0 [113] | 0 [121] |
| 224 | 671 | N [A] | S [G] | 0 [105] | 0.8 [124] | 0 [97] | 1 [113] | 0 [121] |
|  | 672 | N [C] | S [T] | 0 [105] | 0.8 [124] | 0 [97] | 2 [113] | 0 [121] |
| 227 | 681 | L [T] | L [A] | 0.9 [105] | 0 [124] | 0 [97] | 3 [113] | 0 [120] |
| 228 | 683 | S [G] | T [C] | 10 [105] | 13 [124] | 10 [97] | 7 [113] | 5 [121] |
| 232 | 696 | S [A] | S [G] | 0 [105] | 1 [124] | 0 [96] | 2 [113] | 0 [120] |
| 245 | 735 | M [G] | I [A] | 0 [107] | 3 [126] | 0 [95] | 2 [113] | 0 [120] |
| 248 | 744 | E [A] | D [C] | 0 [106] | 1 [126] | 0 [95] | 2 [113] | 0 [119] |
| 249 | 746 | E [A] | A [C] | 0 [106] | 1 [126] | 0 [95] | 2 [113] | 0 [119] |
|  | 747 | E [A] | A [C] | 0 [106] | 1 [126] | 0 [96] | 2 [113] | 0 [119] |
| 250 | 750 | V [T] | V [A] | 0 [106] | 1 [125] | 0 [96] | 2 [112] | 0 [119] |
| 255 | 763 | K [A] | G [G] | 1 [106] | 0.8 [125] | 0 [97] | 0.9 [111] | 0.8 [118] |
|  | 764 | K [A] | R [G] | 92 [106] | 97 [125] | 97 [108] | 98 [111] | 1 [118] |
| 257 | 769 | N [A] | E [G] | 92 [106] | 96 [124] | 97 [95] | 96 [111] | 97 [118] |
|  | 771 | N [T] | E [A] | 92 [106] | 96 [124] | 97 [95] | 96 [111] | 97 [118] |
| 260 | 780 | F [T] | F [C] | 92 [106] | 96 [123] | 97 [95] | 96 [111] | 96 [118] |
| 265 | 795 | Y [T] | Y [C] | 92 [106] | 96 [121] | 97 [92] | 96 [110] | 96 [119] |
| 266 | 798 | D [T] | D [C] | 92 [106] | 96 [121] | 97 [92] | 95 [110] | 95 [120] |
| 270 | 810 | H [T] | H [C] | 92 [100] | 96 [123] | 97 [93] | 98 [110] | 98 [120] |
| 299 | 896 | A [C] | V [T] | 2 [112] | 3 [122] | 2 [93] | 0 [106] | 0 [120] |
| 321 | 962 | Y [A] | S [C] | 0 [108] | 0.8 [120] | 1 [93] | 0 [94] | 0 [121] |
| 335 | 1005 | M [G] | I [T] | 2 [108] | 0.8 [119] | 1 [92] | 1 [97] | 1 [121] |
| 339 | 1017 | G [G] | G [A] | 88 [108] | 88 [120] | 92 [100] | 88 [97] | 89 [121] |
| 343 | 1027 | T [A] | P [C] | 90 [108] | 89 [119] | 93 [100] | 88 [98] | 90 [121] |
| 345 | 1034 | D [A] | G [G] | 90 [108] | 89 [119] | 94 [100] | 92 [98] | 90 [122] |
| 350 | 1048 | V [G] | M [A] | 0 [109] | 0.8 [119] | 0 [99] | 0 [99] | 0 [122] |
| 350 | 1050 | V [A] | V [G] | 90 [109] | 89 [119] | 6 [96] | 93 [99] | 9 [122] |
| 358 | 1074 | N [T] | N [C] | 91 [112] | 86 [121] | 96 [96] | 10 [99] | 10 [123] |
| 373 | 1119 | S [C] | S [T] | 8 [114] | 11 [123] | 3 [96] | 6 [7] | 9 [124] |
| 376 | 1127 | D [A] | V [T] | 0 [114] | 0.7 [126] | 0 [102] | 0 [103] | 0 [124] |
| 393 | 1177 | V [G] | I [A] | 0.8 [116] | 0 [126] | 0 [101] | 0 [104] | 2 [124] |
| 400 | 1198 | A [G] | P [C] | 0.8 [116] | 0 [127] | 0 [101] | 1 [104] | 2 [124] |
|  | 1200 | A [T] | P [G] | 0.8 [116] | 0 [127] | 0 [101] | 2 [104] | 2 [124] |
| 407 | 1221 | F [C] | F [T] | 0.8 [116] | 0 [128] | 0 [101] | 2 [105] | 2 [123] |
| 414 | 1240 | Q [C] | E [G] | 48 [/116] | 42 [128] | 33 [34] | 41 [105] | 41 [123] |
| 434 | 1302 | T [C] | T [T] | 0 [114] | 0 [128] | 1 [99] | 0.8 [122] | 0 [122] |
| 474 | 1420 | L [T] | L [C] | 0.9 [103] | 0.8 [125] | 3 [99] | 0.9 [110] | 0 [1/110] |

The number of samples successfully genotyped per timepoint include: 137 in 1995/96, 141 in 1998/99, 120 in 2005/06, 125 in 2012/13 and 126 in 2015/16. There were a total of 17, 7, 11,3 and 0 sequences with mixed bases in 1995/96, 1998/99, 2005/06, 2012/13 and 2015/16, respectively. Frequency is presented as the percentage of sequences that carried a mutation out of the total number of sequences that had data for that locus [n]. In grey are zero SNP frequencies.

**Supplementary Table 6.** *ubp-1* SNP frequencies

| Codon | Nucleotide | Codon [Nucleotide] | | Mutant Frequency %[n] | | | | |
| --- | --- | --- | --- | --- | --- | --- | --- | --- |
|  |  | Wildtype | Mutant | 1995/96 | 1998/99 | 2005/06 | 2012/13 | 2015/16 |
| 1472 | 4415 | S [C] | Y [A] | 0 [83] | 0.8 [122] | 0.8 [119] | 0 [107] | 0 [108] |
| 1484 | 4450 | D [G] | Y [T] | 0 [83] | 0.7 [126] | 0 [125] | 1.8 [110] | 0 [109] |
| 1499 | 4496 | R [G] | H [A] | 0 [83] | 0.7 [136] | 0 [126] | 0 [112] | 0.9 [110] |
| 1504 | 4511 | E [A] | G [G] | 1.1 [84] | 0 [136] | 0 [127] | 0 [112] | 0 [110] |
| 1505 | 4515 | K [A] | K [G] | 0 [84] | 0 [136] | 0.7 [127] | 0 [112] | 0 [110] |
| 1509 | 4525 | E [GAA] | Del | 2.3 [83] | 3.6 [132] | 5.5 [127] | 4.4 [112] | 4.5 [110] |
| 1514 | 4540 | KNEx2 [AAAAATGAAAAAAACGAA] | 2xKNE [AAAAATGAAAAAAATGAA] | 0 [86] | 9.4 [137] | 5.5 [128] | 11 [112] | 5.4 [112] |
|  |  |  | KNEKND [AAAAATGAAAAAAACGAC] | 0 [86] |  | 0.7 [128] | 0 [112] | 0 [112] |
|  |  |  | KNE [AAAAATGAA] | 0 [86] | 1.4 [137] | 1.5 [128] | 0 [112] | 0 [112] |
|  |  |  | KNE [AAAAACGAA] | 0 [86] |  | 0 [127] | 0.8 [112] | 0.9 [112] |
| 1515 | 4545 | N [T] | N [C] | 0 [86] | 0 [137] | 0 [127] | 0.8 [1] | 0 [111] |
| 1518 | 4554 | N [C] | N [T] | 8.1 [135] | 9.6 [13] | 5.6 [123] | 11 [13] | 5.4 [111] |
| 1519 | 4557 | Y [A] | Y [C] | 0 [87 | 0 [135] | 0.8 [125] | 0 [111] | 0 [110] |
| 1520 | 4558 | KYDx2 [AAATATGACx2] | KYDx4 [AAATATGACx4] | 0 [87 | 11 [137] | 3.1 [/127] | 0.8 [112] | 0 [112] |
|  |  |  | KYDx3 [AAATATGACx3] | 8.0 [87] | 0 [137] | 7.0 [127] | 8.0 [112] | 11 [112] |
|  |  |  | KYGKYD [AAATATGGCAAATATGAC] | 0 [87 | 0.7 [137]] | 0 [127] | 0 [112] | 0 [112] |
|  |  |  | KYD [AAATATGAC] | 10 [87] | 13 [137] | 18 [127] | 16 [112] | 12 [112] |
| 1522 | 4565 | D [A] | G [G] | 0 [87] | 0.7 [137] | 0 [127] | 0 [112] | 0 [111] |
| 1526 | 4576 | KYEx2 [AAATATGAAx2] | KYEKYD[KYEx2] [AAATATGAAAAATATGAC(AAATATGAAx2)] | 0 [87] | 0 [135] | 0.7 [127] | 0 [112] | 0 [112] |
|  |  |  | KYEx3 [AAATATGAAx3] | 11 [87] | 14 [135] | 12 [127] | 10 [112] | 6.3 [112] |
|  |  |  | KYEx3 [AAATACGAA(AAATATGAAx2)] | 0 [87] | 0.7 [135] | 0 [127] | 0 [112] | 0 [112] |
|  |  |  | KYEKYDKYE [AAATATGAAAAATATGACAAATATGAA] | 0 [87] | 0 [135] | 1.5 [127] | 0 [112] | 0 [112] |
|  |  |  | KYEKYV [AAATATGAAAAATATGTA] | 0 [87] | 0.7 [135] | 0 [127] | 0 [112] | 0 [112] |
|  |  |  | KYE [AAATATGAA] | 17 [15] | 11 [135] | 11 [14] | 12 [14] | 17 [112] |
| 1527 | 4581 | Y [T] | Y [C] | 0 [87] | 0.7 [133] | 0 [127] | 0 [112] | 0 [111] |
| 1531 | 4592 | E [A] | V [T] | 0 [72] | 0.8 [122] | 0 [113] | 0 [98] | 0 [92] |
| 1532 | 4594 | KYD [AAATATGAT] | KYDx2 [(2xAAATATGAT)] | 1.1 [85] | 0 [135] | 0 [126] | 0 [111] | 0 [111] |
| 1575 | 4724 | K [A] | R [G] | 0 [86] | 0 [135] | 0.7 [126] | 0 [105] | 0 [108] |

The number of samples successfully genotyped per timepoint include: 85 in 1995/96, 137 in 1998/99, 128 in 2005/06, 112 in 2012/13 and 85 in 2015/16. No sequences with mixed bases were identified. Frequency is presented as the percentage of sequences that carried a mutation out of the total number of sequences that had data for that locus [n]. In grey are zero SNP frequencies

**Supplementary Table 7.** *nfs* SNP frequencies

| Codon | Nucleotide | Codon [Nucleotide] | | Mutant Frequency % [n] | | | | | |
| --- | --- | --- | --- | --- | --- | --- | --- | --- | --- |
|  |  | Wildtype | Mutant | 1995/96 | 1998/99 | 2005/06 | 2012/13 | 2015/16 | 2017/18 |
| 44 | 131 | S [C] | C [G] | 0 [73] | 0 [64] | 2.0 [48] | 4.4 [68] | 6.1 [97] | 4.2 [70] |
| 62 | 185 | S [G] | N [A] | 37 [74] | 33 [64] | 36 [47] | 28 [73] | 25 [99] | 20 [71] |
| 65 | 193 | K [A] | Q [C] | 62 [74] | 66 [64] | 40 [47] | 70 [74] | 74 [99] | 80 [70] |
| 67 | 200 | E [A] | G [G] | 62 [74] | 66 [64] | 40 [47] | 70 [74] | 74 [79] | 80 [70] |
|  | 201 | E [A] | G [T] | 62 [74] | 66 [64] | 40 [47] | 70 [74] | 74 [79] | 80 [70] |
| 110 | 328 | Q [C] | E [G] | 5.4 [74] | 10 [65] | 8 [50] | 1.2 [78] | 16 [79] | 5.7 [70] |
| 116 | 347 | S [G] | I [T] | 0 [74] | 1.5 [66] | 0 [51] | 0 [79] | 0 [100] | 98 [70] |
| 119 | 355 | G [G] | C [T] | 0 [74] | 3.0 [67] | 0 [51] | 1.2 [79] | 0 [100] | 0 [70] |
| 120 | 359 | S [G] | I [T] | 14 [74] | 27 [66] | 37 [51] | 30 [79] | 41 [100] | 32 [70] |
| 126 | 376 | P [C] | S [T] | 0 [74] | 0 [65] | 1.9 [51] | 0 [79] | 0 [100] | 0 [70] |
| 130 | 389 | E [A] | G [G] | 0 [74] | 0 [65] | 0 [51] | 1.2 [79] | 0 [100] | 0 [70] |
|  | 390 | E [G] | D [C] | 13 [74] | 18 [65] | 15 [51] | 18 [79] | 25 [25] | 22 [70] |
| 188 | 562 | E [G] | K [A] | 0 [24] | 0 [57] | 0 [44] | 0 [60] | 0 [92] | 5.9 [67] |
| 274 | 822 | G [T] | G [C] | 0 [76] | 0 [66] | 0 [57] | 0 [82] | 0 [100] | 1.4 [70] |
| 283 | 847 | N [A] | Y [T] | 0 [76] | 0 [66] | 0 [58] | 0 [82] | 0 [100] | 1.4 [70] |
| 288 | 864 | N [T] | N [C] | 0 [76] | 0 [66] | 1.7 [58] | 0 [82] | 0 [100] | 0 [70] |
| 308 | 922 | E [G] | Q [C] | 0 [76] | 0 [66] | 0 [57] | 0 [82] | 0 [100] | 1.4 [70] |
| 334 | 1002 | I [C] | I [A] | 1.3 [75] | 0 [66] | 0 [56] | 0 [82] | 0 [100] | 0 [70] |
| 338 | 1013 | K [A] | R [G] | 0 [75] | 0 [66] | 0 [56] | 0 [82] | 0 [100] | 1.4 [70] |
| 379 | 1137 | G [C] | G [T] | 0 [74] | 0 [66] | 0 [54] | 0 [82] | 0 [100] | 1.4 [70] |
| 409 | 1225 | N [A] | Y [T] | 0 [74] | 0 [66] | 0 [53] | 0 [82] | 0 [102] | 2.9 [69] |

The number of samples successfully genotyped per timepoint include: 81 in 1995/96, 71 in 1998/99, 73 in 2005/06, 86 in 2012/13, 114 in 2015/16 and 77 in 2017/18. There were a total of 4, 3, 3, 4, 11 and 7 sequences with mixed bases in 1995/96, 1998/99, 2005/06, 2012/13, 2015/16 and 2017/18, respectively. Frequency is presented as the percentage of sequences that carried a mutation out of the total number of sequences that had data for that locus [n]. In grey are zero SNP frequencies.

**Supplementary Table 8.** *serine-tRNA ligase* SNP frequencies

| Codon | Nucleotide | Codon [Nucleotide] | | | Mutant Frequency %[n] | | | | |
| --- | --- | --- | --- | --- | --- | --- | --- | --- | --- |
|  |  | Wildtype | Mutant | 1995/96 | | 1998/99 | 2005/06 | 2012/13 | 2015/16 |
| 31 | 92 | N [A] | S [G] | 0 [95] | | 0 [120] | 0 [105] | 0.8 [114] | 3 [127] |
| 71 | 211 | D [G] | N [A] | 2 [105] | | 0 [133] | 0 [121] | 0 [121] | 0 [142] |
| 84 | 250 | L [T] | V [G] | 3 [106] | | 3 [133] | 5 [120] | 4 [122] | 2 [142] |
| 86 | 258 | I [A] | M [G] | 0 [106] | | 0 [133] | 0 [121] | 0 [122] | 1 [142] |
| 88 | 264 | E [A] | D [T] | 0 [106] | | 6 [133] | 0.8 [121] | 1 [122] | 2 [142] |
| 94 | 280 | Q [C] | E [G] | 0 [107] | | 0 [133] | 0 [121] | 0.8 [122] | 2 [142] |
| 133 | 399 | T [A] | T [G] | 0 [106] | | 2 [132] | 0.8 [121] | 0 [120] | 0.7 [142] |
| 140 | 418 | L [C] | F [T] | 0 [105] | | 0.7 [132] | 0 [118] | 0 [119] | 0 [142] |
| 155 | 463 | V [G] | L [T] | 0 [105] | | 0 [133] | 0 [120] | 1 [118] | 0 [142] |
| 175 | 524 | I [T] | K [A] | 0 [95] | | 0 [128] | 0.9 [108] | 0 [114] | 0 [139] |
| 199 | 596 | A [C] | V [T] | 0 [91] | | 0 [116] | 0 [190] | 0 [113] | 0.7 [134] |
| 221 | 662 | A [C] | G [G] | 0 [94] | | 0.8 [124] | 0.9 [111] | 1 [120] | 0.7 [138] |
| 267 | 799 | T [A] | P [C] | 0 [93] | | 0 [126] | 0 [113] | 5 [118] | 1 [136] |
| 284 | 851 | A [C] | V [T] | 0 [95] | | 0.7 [128] | 0 [110] | 0 [117] | 0 [136] |
| 318 | 952 | E [G] | K [A] | 0 [94] | | 0.7 [130] | 0 [107] | 0 [115] | 0 [139] |
| 372 | 1116 | F [F] | F [T] | 1 [89] | | 0 [127] | 0 [107] | 0 [115] | 0 [139] |
| 417 | 1249 | S [T] | A [G] | 0 [92] | | 1 [126] | 0 [105] | 0 [117] | 0.7 [139] |
| 430 | 1290 | N [T] | N [C] | 0 [92] | | 1 [129] | 0 [107] | 0 [120] | 0 [139] |
| 528 | 1584 | Y [C] | Y [T] | 0 [99] | | 0 [126] | 0 [110] | 1 [101] | 0 [139] |

The number of samples successfully genotyped per timepoint include: 118 in 1995/96, 136 in 1998/99, 130 in 2005/06, 123 in 2012/13 and 118 in 2015/16. No sequences with mixed bases were identified. Frequency is presented as the percentage of sequences that carried a mutation out of the total number of sequences that had data for that locus [n]. In grey are zero SNP frequencies.

**Supplementary Table 9.** *ap2-mu haplotype* frequencies

| Haplotype | Frequency % [n] | | | | |
| --- | --- | --- | --- | --- | --- |
|  | 1995/96 | 1998/99 | 2005/06 | 2012/13 | 2015/16 |
| TRSKT[N7][Kx1]AG[N5][N4]AFI* | 52.1 [46] | 56.1 [73] | 43.7 [16] | 39.4 [38] | 40.3 [62] |
| TRNKT[N7][Kx1]AG[N5][N4]AFI | 6.5 [46] | 4.1 [73] | 0 [16] | 13.1 [38] | 11.2 [62] |
| TRSKT[N7][Kx1]AG[N5][N4]ALI | 6.5 [46] | 0 [73] | 0 [16] | 5.2 [38] | 3.2 [62] |
| TRSKT[N9][Kx1]AG[N5][N4]AFI | 6.5 [46] | 5.4 [73] | 6.2 [16] | 0 [38] | 9.6 [62] |
| TRSKT[N7][Kx1]AG[N5][N5]AFI | 4.3 [46] | 0 [73] | 0 [16] | 0 [38] | 3.2 [62] |
| TRSKT[N7][Kx1]AG[N6][N4]AFI | 4.3 [46] | 2.7 [73] | 6.2 [16] | 0 [38] | 0 [62] |
| TRSKT[N8][Kx1]AG[N5][N4]AFI | 4.3 [46] | 8.2 [73] | 18.7 [16] | 7.8 [38] | 11.2 [62] |
| TRSTT[N7][Kx1]AG[N5][N4]AFI | 4.3 [46] | 2.7 [73] | 0 [16] | 5.2 [38] | 1.6 [62] |
| TRNKT[N8][Kx1]AG[N5][N4]ALI | 2.1 [46] | 0 [73] | 0 [16] | 0 [38] | 0 [62] |
| TRNKT[N8][Kx1]TG[N5][N4]AFI | 2.1 [46] | 0 [73] | 0 [16] | 0 [38] | 0 [62] |
| TRSKR[N7][Kx1]AG[N6][N4]AFI | 2.1 [46] | 0 [73] | 0 [16] | 0 [38] | 0 [62] |
| TRSTT[N7][Kx2]AG[N5][N4]AFI | 2.1 [46] | 0 [73] | 0 [16] | 0 [38] | 0 [62] |
| TRSTT[N9][Kx1]AG[N5][N4]AFI | 2.1 [46] | 0 [73] | 0 [16] | 0 [38] | 1.6 [62] |
| TRNKT[N7][Kx1]AG[N6][N4]AFI | 0 [46] | 2.7 [73] | 0 [16] | 0 [38] | 0 [62] |
| TRSTT[N8][Kx1]AG[N5][N4]AFI | 0 [46] | 2.7 [73] | 0 [16] | 0 [38] | 3.2 [62] |
| TKSKT[N9][Kx1]AG[N5][N4]AFI | 0 [46] | 1.3 [73] | 6.2 [16] | 0 [38] | 0 [62] |
| TRNKT[N7][Kx1]AG[N5][N5]AFI | 0 [46] | 1.3 [73] | 0 [16] | 0 [38] | 0 [62] |
| TRNKT[N8][Kx1]AG[N5][N4]AFI | 0 [46] | 1.3 [73] | 6.2 [16] | 0 [38] | 1.6 [62] |
| TRNKT[N8][Kx1]AG[N5][N4]AFT | 0 [46] | 1.3 [73] | 0 [16] | 0 [38] | 0 [62] |
| TRNTT[N7][Kx1]AG[N5][N4]AFI | 0 [46] | 1.3 [73] | 0 [16] | 2.6 [38] | 4.8 [62] |
| TRSKT[N10][Kx1]AG[N5][N4]AFI | 0 [46] | 1.3 [73] | 0 [16] | 0 [38] | 0 [62] |
| TRSKT[N6][Kx2]AG[N5][N4]TFI | 0 [46] | 0 [73] | 0 [16] | 0 [38] | 0 [62] |
| TRSKT[N7][Kx1]AG[N7][N4]AFI | 0 [46] | 1.3 [73] | 0 [16] | 0 [38] | 0 [62] |
| TRSKT[N7][Kx2]AG[N6][N4]AFI | 0 [46] | 1.3 [73] | 0 [16] | 2.6 [38] | 0 [62] |
| TRSKT[N9][Kx1]AD[N5][N4]AFI | 0 [46] | 1.3 [73] | 0 [16] | 0 [38] | 0 [62] |
| TRSTT[N7][Kx1]AG[N7][N4]AFI | 0 [46] | 1.3 [73] | 0 [16] | 0 [38] | 0 [62] |
| ARSKT[N8][Kx1]AG[N5][N4]AFI | 0 [46] | 0 [73] | 6.2 [16] | 0 [38] | 0 [62] |
| TRNKT[N9][Kx1]AG[N5][N4]AFI | 0 [46] | 0 [73] | 6.2 [16] | 0 [38] | 0 [62] |
| TRNKT[N7][Kx2]AG[N5][N4]AFI | 0 [46] | 0 [73] | 0 [16] | 10.5 [38] | 0 [62] |
| TRSKT[N7][Kx1]AG[N5][N4]TFI | 0 [46] | 0 [73] | 0 [16] | 2.6 [38] | 0 [62] |
| TRSKT[N7][Kx2]AG[N5][N4]AFI | 0 [46] | 0 [73] | 0 [16] | 2.6 [38] | 0 [62] |
| TRSKT[N8][Kx1]AG[N6][N4]AFI | 0 [46] | 0 [73] | 0 [16] | 2.6 [38] | 0 [62] |
| TRSTT[N8][Kx1]AG[N6][N4]AFI | 0 [46] | 0 [73] | 0 [16] | 2.6 [38] | 0 [62] |
| TRSTT[N9][Kx1]AG[N6][N4]AFI | 0 [46] | 0 [73] | 0 [16] | 2.6 [38] | 0 [62] |
| TRNTT[N9][Kx1]AG[N5][N4]AFI | 0 [46] | 0 [73] | 0 [16] | 0 [38] | 3.2 [62] |
| TRSKT[N10][Kx1]AG[N5][N6]AFI | 0 [46] | 0 [73] | 0 [16] | 0 [38] | 1.6 [62] |
| TRSKT[N9][Kx1]AG[N6][N4]AFI | 0 [46] | 0 [73] | 0 [16] | 0 [38] | 1.6 [62] |
| TRSTT[N7][Kx1]AG[N6][N4]AFI | 0 [46] | 0 [73] | 0 [16] | 0 [38] | 1.6 [62] |

The following were used to define haplotypes: 14 polymorphic sites, including codons 144, 146, 160, 199, 207, 227, 234, 236, 315, 320, 327, 337, 437, 498. The haplotype marked with an * indicate the 3D7 haplotype. Frequency is presented as the percentage of sequences that contributed to the haplotype out of the total number of sequences [n]. In grey are zero SNP frequencies.

**Supplementary Table 10.** *falcipain-2a haplotype* frequencies

| Haplotype | Frequency % [n] | | | | |
| --- | --- | --- | --- | --- | --- |
|  | 1995/96 | 1998/99 | 2005/06 | 2012/13 | 2015/16 |
| NAQSRNEGTIKDFMNNENSMEEREAYMPGVDVAQ | 22.8 [83] | 31.7 [104] | 37.3 [67] | 45.7 [83] | 33.6 [104] |
| NAQSRNEGTIKDFMNNENSMEEREAYMPGVDVAE | 20.4 [83] | 14.4 [104] | 10.4 [67] | 14.4 [83] | 15.3 [104] |
| HIQSRNEGTIKDFMNNENSMEEREAYMPGVDVAQ | 4.8 [83] | 7.6 [104] | 10.4 [67] | 3.6 [83] | 5.7 [104] |
| NAQFRNEGTIKDFMNNENSMEEREAYMPGVDVAQ | 4.8 [83] | 1.9 [104] | 4.4 [67] | 1.2 [83] | 4.8 [104] |
| NAQSRNEGTIKDFMNNENTMEEREAYMTDVDVAE | 4.8 [83] | 6.7 [104] | 1.4 [67] | 2.4 [83] | 1.9 [104] |
| NAQSRNEGTIKDFMNNENSMEEKNAYMPGVDVAE | 3.6 [83] | 0 [104] | 1.4 [67] | 0 [83] | 0 [104] |
| NAQSRNEGTIKDFMNNENSMEEKNAYMPGVDVAQ | 3.6 [83] | 1.9 [104] | 0 [67] | 0 [83] | 0.9 [104] |
| NAQSRNEGTIKDFMNNENTMEEREAYMPGVDVAQ | 3.6 [83] | 1.9 [104] | 2.9 [67] | 1.2 [83] | 0 [104] |
| HIQSRNEGTIKDFMNNENTMEEREAYMPGVDVAQ | 2.4 [83] | 0 [104] | 1.4 [67] | 0 [83] | 0 [104] |
| NAQFRNEGTIKDFMNNENSMEEREAYMPGVDVAE | 2.4 [83] | 0.9 [104] | 2.9 [67] | 0 [83] | 0 [104] |
| HAHSRNEGTIKDFMNNENSMEEREAYMPGVDVAQ | 1.2 [83] | 0 [104] | 1.4 [67] | 1.2 [83] | 0 [104] |
| HIQSRNEGTIKDFMNNENSMEEREAYMPGVDVAE | 1.2 [83] | 0 [104] | 0 [67] | 0 [83] | 1.9 [104] |
| HIQSRNEGTIKDFMNNENSMEEREAYMTDVDVAE | 1.2 [83] | 0 [104] | 0 [67] | 0 [83] | 0 [104] |
| NAHFRNEGTIKDFMNNENSMEEREAYMPGVDVAE | 1.2 [83] | 0.9 [104] | 0 [67] | 0 [83] | 0 [104] |
| NAHFRNEGTIKDFMNNENSMEEREAYMTDVDVAE | 1.2 [83] | 0 [104] | 0 [67] | 0 [83] | 0 [104] |
| NAHSRNEGKIKDFMNNENSMEEREAYMPGVDVAE | 1.2 [83] | 0 [104] | 0 [67] | 1.2 [83] | 0 [104] |
| NAHSRNEGTIKDFMNNENSMEEKNAYMPGVDVAQ | 1.2 [83] | 0 [104] | 0 [67] | 0 [83] | 0 [104] |
| NAHSRNEGTIKDFMNNENSMEEREAYIPGVDVAE | 1.2 [83] | 0 [104] | 0 [67] | 0 [83] | 0 [104] |
| NAHSRNEGTIKDFMNNENSMEEREAYMPGVDVAE | 1.2 [83] | 4.8 [104] | 2.9 [67] | 3.6 [83] | 7.6 [104] |
| NAHSRNEGTIKDFMNNENSMEEREAYMPGVDVAQ | 1.2 [83] | 0.9 [104] | 0 [67] | 1.2 [83] | 0.9 [104] |
| NAHSRNEGTIKDFMNNENTMEEREAYMPGVDVAE | 1.2 [83] | 0 [104] | 2.9 [67] | 2.4 [83] | 0 [104] |
| NAQFRNEGTIKDFMNNENSMEEGEAYMPGVDVAQ | 1.2 [83] | 0 [104] | 0 [67] | 0 [83] | 0 [104] |
| NAQFRNEGTIKDFMNNENSMEEREAYIPGVDVAQ | 1.2 [83] | 0 [104] | 0 [67] | 0 [83] | 0 [104] |
| NAQFRNEGTIKDFMNNENSMEEREAYMTDVDVAE | 1.2 [83] | 0.9 [104] | 0 [67] | 0 [83] | 0 [104] |
| NAQSRNEGTIKDFMKNENSMEEREAYMPGVDVAQ | 1.2 [83] | 0 [104] | 0 [67] | 0 [83] | 0 [104] |
| NAQSRNEGTIKDFMKNENSMEEREAYMTDVDVAE | 1.2 [83] | 1.9 [104] | 1.4 [67] | 1.2 [83] | 1.9 [104] |
| NAQSRNEGTIKDFMNNENSMEEREAYIPGVDVAQ | 1.2 [83] | 0 [104] | 0 [67] | 0 [83] | 0.9 [104] |
| NAQSRNEGTIKDFMNNENSMEEREAYMTDVDVAE | 1.2 [83] | 0 [104] | 1.4 [67] | 2.4 [83] | 2.8 [104] |
| NAQSRNEGTIKDFMNNENSMEEREVYMPGVDVAE | 1.2 [83] | 0 [104] | 0 [67] | 0 [83] | 0 [104] |
| NAQSRNEGTIKDFMNNENTMEEGEAYMPGVDVAE | 1.2 [83] | 0.9 [104] | 0 [67] | 1.2 [83] | 0 [104] |
| NAQSRNEGTIKDFTNNENSMEEREAYMPGVDVAQ | 1.2 [83] | 0 [104] | 0 [67] | 0 [83] | 0 [104] |
| NAQSRNEGTLKDFMNNENSMEEREAYMPGVDVAQ | 1.2 [83] | 0 [104] | 1.4 [67] | 0 [83] | 0 [104] |
| NAQSRNEDTIKDFMNNENSMEEREVYMPGVDVAE | 0 [83] | 3.8 [104] | 0 [67] | 0 [83] | 0 [104] |
| NAHSRNVGTIKDFMNNENSMEEREAYMPGVDVAQ | 0 [83] | 2.8 [104] | 0 [67] | 0 [83] | 0.9 [104] |
| NAHSRNEGTIKDFMNNENSIDAKNAYMTDVDVAE | 0 [83] | 1.9 [104] | 0 [67] | 0 [83] | 0 [104] |
| HAQSRNEGTIKDFMNNENSMEEREAYMPGVDVAQ | 0 [83] | 0.9 [104] | 0 [67] | 0 [83] | 0 [104] |
| HVQSRNEGTIKDFMNNENSMEEREAYMPGVDVAQ | 0 [83] | 0.9 [104] | 1.4 [67] | 0 [83] | 0 [104] |
| NAHSRNEGTIKDFMNNENSMEEREAYMPGMDVAE | 0 [83] | 0.9 [104] | 0 [67] | 0 [83] | 0 [104] |
| NAQFRNEGTIKDFMNNENSMEEREAYIPGVDVAE | 0 [83] | 0.9 [104] | 0 [67] | 0 [83] | 0 [104] |
| NAQSRNEGTIKDFMKNENSMEEREAYMPGVDVAE | 0 [83] | 0.9 [104] | 0 [67] | 0 [83] | 0.9 [104] |
| NAQSRNEGTIKDFMNKESTIEEREAYMPGVDVAQ | 0 [83] | 0.9 [104] | 0 [67] | 0 [83] | 0 [104] |
| NAQSRNEGTIKDFMNNENSMEEKEAYMPGVDVAQ | 0 [83] | 0.9 [104] | 0 [67] | 0 [83] | 0 [104] |
| NAQSRNEGTIKDFMNNENSMEEREASMPGVDVAE | 0 [83] | 0.9 [104] | 1.4 [67] | 0 [83] | 0 [104] |
| NAQSRNEGTIKDFMNNENSMEEREAYMPGVVVAE | 0 [83] | 0.9 [104] | 0 [67] | 0 [83] | 0 [104] |
| NAQSRNEGTIKDFMNNENTMEEREAYMPGVDVAE | 0 [83] | 0.9 [104] | 1.4 [67] | 1.2 [83] | 0 [104] |
| NAQSRNEGTIKDFTNNENTMEEREAYMPGVDVAE | 0 [83] | 0.9 [104] | 0 [67] | 0 [83] | 0 [104] |
| NAQSRNEGTIKDYMNNENSMEEREAYMPGVDVAE | 0 [83] | 0.9 [104] | 0 [67] | 0 [83] | 0 [104] |
| NVHFRNEGTIKDFMNNENSMEEREAYMPGVDVAQ | 0 [83] | 0.9 [104] | 0 [67] | 0 [83] | 0 [104] |
| NVQSRNEGTIKDFMNNENSMEEREAYMPGVDVAQ | 0 [83] | 0.9 [104] | 0 [67] | 0 [83] | 0 [104] |
| HIHSRNEGTIKDFMNNENSMEEREAYMPGVDVAE | 0 [83] | 0 [104] | 2.9 [67] | 1.2 [83] | 0.9 [104] |
| HIHSRNEGTIKDFMNNENSMEEREAYMPGVDVAQ | 0 [83] | 0 [104] | 1.4 [67] | 2.4 [83] | 0.9 [104] |
| NAHSRDEGTIKDFMNNENSMEEREAYMPGVDVAQ | 0 [83] | 0 [104] | 1.4 [67] | 0 [83] | 0 [104] |
| NAQSRNEGTIENFMNNENSMEEREAYMPGVDVAQ | 0 [83] | 0 [104] | 1.4 [67] | 1.2 [83] | 0 [104] |
| NAQSRNEGTIKDFMNNENTMEEREAYMTGVDVAE | 0 [83] | 0 [104] | 1.4 [67] | 1.2 [83] | 0 [104] |
| NAQSRNVGTIKDFMNNENSMEEREAYMPGVDVAQ | 0 [83] | 0 [104] | 1.4 [67] | 0 [83] | 0 [104] |
| NAQSRNEGTIKDFMNNENSIDAKNAYMTGVDVAE | 0 [83] | 0 [104] | 0 [67] | 2.4 [83] | 0 [104] |
| HIQSRNEGTIKDFMNNQNSMEEREAYMPGVDVAQ | 0 [83] | 0 [104] | 0 [67] | 1.2 [83] | 5.7 [104] |
| NAQSKNEGTIKDFMNNENSMEEREAYMPGVDVAE | 0 [83] | 0 [104] | 0 [67] | 1.2 [83] | 0 [104] |
| NAQSRNEGTIKDFMNNENSIEEREAYMPGVDVAQ | 0 [83] | 0 [104] | 0 [67] | 1.2 [83] | 0 [104] |
| NAQSRNEGTIKDFMNNENSMEEREAYIPGVDVAE | 0 [83] | 0 [104] | 0 [67] | 1.2 [83] | 0 [104] |
| NAQSRNEGTIKDFMNNENSMEEREAYMTDVDVAQ | 0 [83] | 0 [104] | 0 [67] | 1.2 [83] | 0 [104] |
| NAQSRNEGTIKDFMNNENSMEERNAYMTDVDVAE | 0 [83] | 0 [104] | 0 [67] | 1.2 [83] | 0.9 [104] |
| NAQSRNEGTIKDFMNNENSMEEREAYMPGVDIPE | 0 [83] | 0 [104] | 0 [67] | 0 [83] | 2.8 [104] |
| HAQSRNEGTIKDFMKNENSMEEREAYMPGVDVAQ | 0 [83] | 0 [104] | 0 [67] | 0 [83] | 0.9 [104] |
| HAQSRNEGTIKDFMNNENSMEEREAYMPGVDVAE | 0 [83] | 0 [104] | 0 [67] | 0 [83] | 0.9 [104] |
| NAHSRNEGTIKDFMNNENTMEEREAYMTGVDVAE | 0 [83] | 0 [104] | 0 [67] | 0 [83] | 0.9 [104] |
| NAQSKNEGTIKDFMNNENSMEEREAYMPGVDVAQ | 0 [83] | 0 [104] | 0 [67] | 0 [83] | 0.9 [104] |
| NAQSRNEGTIKDFMKNENSMEEREAYMPDVDVAE | 0 [83] | 0 [104] | 0 [67] | 0 [83] | 0.9 [104] |
| NAQSRNEGTIKDFMNNENTMEEGEAYMPGVDVAQ | 0 [83] | 0 [104] | 0 [67] | 0 [83] | 0.9 [104] |
| NAQSRNEGTIKDFMNNENTMEEREAYMTDVDVAQ | 0 [83] | 0 [104] | 0 [67] | 0 [83] | 0.9 [104] |
| NVHFRNEGTIKDFMNNENSMEEREAYIPGVDVAQ | 0 [83] | 0 [104] | 0 [67] | 0 [83] | 0.9 [104] |

The following were used to define haplotypes: 35 polymorphic sites, including codons 4, 8, 15, 59, 60, 86, 114, 115, 126, 134, 140, 143, 144, 150, 167, 173, 204, 210, 224, 228, 245, 248, 249, 255, 257, 299, 321, 335, 343, 345, 350, 376, 393, 400, 414. The3D7 haplotype was not identified. Frequency is presented as the percentage of sequences that contributed to the haplotype out of the total number of sequences [n]. In grey are zero SNP frequencies.

**Supplementary Table 11.** *ubp-1* haplotype frequencies

| Haplotype | Frequency % [n] | | | | |
| --- | --- | --- | --- | --- | --- |
|  | 1995/96 | 1998/99 | 2005/06 | 2012/13 | 2015/16 |
| SDREE[KNEx2][KYDx2]D[KYEx2]E[KYDx1]* | 66.6 [66] | 62.2 [106] | 58.2 [103] | 62.9 [89] | 70.9 [86] |
| SDREE[KNEx2][KYDx1]D[KYEx2]E[KYDx1] | 10.6 [66] | 11.3 [106] | 16.5 [103] | 14.6 [89] | 12.7 [86] |
| SDREE[KNEx2][KYDx2]D[KYEx3]E[KYDx1] | 9.0 [66] | 10.3 [106] | 8.7 [103] | 10.1 [89] | 4.6 [86] |
| SDREE[KNEx2][KYDx3]D[KYEx2]E[KYDx1] | 4.5 [66] | 2.8 [106] | 3.8 [103] | 2.2 [89] | 2.3 [86] |
| SDRE-[KNEx2][KYDx2]D[KYEx3]E[KYDx1] | 1.5 [66] | 1.8 [106] | 0.9 [103] | 0 [89] | 0 [86] |
| SDRE-[KNEx2][KYDx3]D[KYEx2]E[KYDx1] | 1.5 [66] | 0 [106] | 0 [103] | 1.1 [89] | 3.4 [86] |
| SDREE[KNEx2][KYDx1]D[KYEx3]E[KYDx1] | 1.5 [66] | 0.9 [106] | 0 [103] | 1.1 [89] | 1.1 [86] |
| SDREE[KNEx2][KYDx2]D[KYEx2]E[KYDx2] | 1.5 [66] | 0 [106] | 0 [103] | 0 [89] | 0 [86] |
| SDREE[KNEx2][KYDx3]D[KYEx3]E[KYDx1] | 1.5 [66] | 0.9 [106] | 1.9 [103] | 0 [89] | 0 [86] |
| SDRGE[KNEx2][KYDx2]D[KYEx2]E[KYDx1] | 1.5 [66] | 0 [106] | 0 [103] | 0 [89] | 0 [86] |
| SDREE[KNEx2][KYDx2]D[KYEx1]E[KYDx1] | 0 [66] | 1.8 [106] | 0 [103] | 0 [89] | 0 [86] |
| SDREE[KNEx2][KYDx4]D[KYEx2]E[KYDx1] | 0 [66] | 1.8 [106] | 1.9 [103] | 0 [89] | 0 [86] |
| SDRE-[KNEx2][KYDx1]D[KYEx2]E[KYDx1] | 0 [66] | 0.9 [106] | 1.9 [103] | 3.3 [89] | 0 [86] |
| SDREE[KNEx1][KYDx3]D[KYEx2]E[KYDx1] | 0 [66] | 0.9 [106] | 0 [103] | 0 [89] | 1.1 [86] |
| SDREE[KNEx2][KYDx2]D[KYEKYV]V[KYDx1] | 0 [66] | 0.9 [106] | 0 [103] | 0 [89] | 0 [86] |
| SDREE[KNEx2][KYGKYD]G[KYEx3]E[KYDx1] | 0 [66] | 0.9 [106] | 0 [103] | 0 [89] | 0 [86] |
| SYREE[KNEx2][KYDx2]D[KYEx2]E[KYDx1] | 0 [66] | 0.9 [106] | 0 [103] | 2.2 [89] | 0 [86] |
| YDREE[KNEx1][KYDx2]D[KYEx3]E[KYDx1] | 0 [66] | 0.9 [106] | 0.9 [103] | 0 [89] | 0 [86] |
| SDRE-[KNEx2][KYDx4]D[KYEx2]E[KYDx1] | 0 [66] | 0 [106] | 1.9 [103] | 0 [89] | 0 [86] |
| SDREE[KNEx2][KYDx2]D[KYEKYDKYE]E[KYDx1] | 0 [66] | 0 [106] | 1.9 [103] | 0 [89] | 0 [86] |
| SDREE[KNEx1][KYDx2]D[KYEx3]E[KYDx1] | 0 [66] | 0 [106] | 0.9 [103] | 1.1 [89] | 0 [86] |
| SDRE-[KNEx2][KYDx2]D[KYEx2]E[KYDx1] | 0 [66] | 0 [106] | 0 [103] | 1.1 [89] | 2.3 [86] |
| SDHEE[KNEx2][KYDx2]D[KYEx3]E[KYDx1] | 0 [66] | 0 [106] | 0 [103] | 0 [89] | 1.1 [86] |

The following were used to define haplotypes: 12 polymorphic sites, including codons 1472, 1484, 1499, 1504, 1508, 1514, 1520, 1522, 1526, 1531, 1532 and 1575. The haplotype marked with an * indicate the 3D7 haplotype. "-" in some of the haplotypes indicates a deletion. Frequency is presented as the percentage of sequences that contributed to the haplotype out of the total number of sequences [n]. In grey are zero SNP frequencies.

**Supplementary Table 12.** *crt*, *mdr1* and *dhps* haplotype frequencies

| Gene | Haplotype | Frequency % [n] | | | | | | |
| --- | --- | --- | --- | --- | --- | --- | --- | --- |
|  |  | 1995/96 | 1998/99 | 2005/06 | 2012/13 | 2015/16 | 2017/18 |  |
| *crt* | CVMNK* | 37.9 [79] | 6.8 [103] | 49.0 [102] | 81.7 [82] | 96.7 [91] | 98.8 [90] |  |
|  | CVIET | 62.0 [79] | 93.2 [103] | 50.9 [102] | 18.2 [82] | 3.3 [91] | 1.1 [90] |  |
| *mdr1* | NYD* | 14.2 [56] | 12.3 [81] | 12.7 [47] | 64.5 [48] | 40 [60] | 55.7 [70] |  |
|  | NFD | 28.5 [56] | 7.4 [81] | 12.7 [47] | 33.3 [48] | 55 [60] | 41.4 [70] |  |
|  | YFY | 1.7 [56] | 0 [81] | 0 [47] | 0 [48] | 0 [60] | 0 [70] |  |
|  | YYD | 19.6 [56] | 16.0 [81] | 31.9 [47] | 2.0 [48] | 3.3 [60] | 1.4 [70] |  |
|  | YYY | 35.7 [56] | 56.7 [81] | 21.2 [47] | 0 [48] | 0 [60] | 0 [70] |  |
|  | NFY | 0 [56] | 6.1 [81] | 12.7 [47] | 0 [48] | 0 [60] | 1.4 [70] |  |
|  | NYY | 0 [56] | 1.2 [81] | 4.2 [47] | 0 [48] | 1.6 [60] | 0 [70] |  |
|  | YFD | 0 [56] | 0 [81] | 4.2 [47] | 0 [48] | 0 [60] | 0 [70] |  |
| *dhps* | SGEA* | 10.7 [84] | 31.6 [115] | 84.2 [57] | 76 [25] | 85.7 [21] | NA |  |
|  | SAKA | 66.6 [84] | 59.8 [115] | 14.0 [57] | 16 [25] | 0 [21] | NA |  |
|  | SGKA | 22.6 [84] | 5.9 [115] | 1.7 [57] | 8 [25] | 9.5 [21] | NA |  |
|  | AAKA | 0 [84] | 2.5 [115] | 0 [57] | 0 [25] | 0 [21] | NA |  |
|  | SGEG | 0 [84] | 0 [115] | 0 [57] | 0 [25] | 4.7 [21] | NA |  |

The following were used to define haplotypes: crt - 3 polymorphic sites, including codons 74, 75 and 76. mdr1 - 3 polymorphic sites, including codons 86, 184 and 1246. dhps - 4 polymorphic sites, including codons 436, 437, 540 and 581. The haplotypes marked with an * indicate the 3D7 haplotype. For dhps, there was no data for the 2017/18 timepoint as it was not genotyped . Frequency is presented as the percentage of sequences that contributed to the haplotype out of the total number of sequences [n]. In grey are zero SNP frequencies.

**Supplementary Table 13.** *nfs* haplotype frequencies

| **Haplotype** | **Frequency % [n]** | | | | | |
| --- | --- | --- | --- | --- | --- | --- |
|  | **1995/96** | **1998/99** | **2005/06** | **2012/13** | **2015/16** | **2017/18** |
| SNQGQSGIPEENEKN* | 27.27[22] | 7.14 [56] | 17.07 [41] | 15.25 [59] | 9.78 [92] | 8.96 [67] |
| SSKEQSGSPDENEKN | 22.73[22] | 3.57 [56] | 2.44 [41] | 5.08 [59] | 4.35 [92] | 2.99 [67] |
| SNQGQSGSPDENEKN | 13.64 [22] | 10.71 [56] | 0 [41] | 6.78 [59] | 3.26 [92] | 7.46 [67] |
| SNQGQSGSPEENEKN | 13.64 [22] | 37.5 [56] | 31.71 [41] | 37.29 [59] | 28.26 [92] | 35.82 (67) |
| SNQGESGIPEENEKN | 9.09 [22] | 7.14 [56] | 4.88 [41] | 0 [59] | 6.52 [92] | 1.49 [67] |
| SNQGQSGIPDENEKN | 9.09 [22] | 3.57 [56] | 2.44 [41] | 8.47 [59] | 13.04 (12) | 4.48 [67] |
| SSKEQSGSPEENEKN | 4.55 [22] | 10.71 [56] | 12.2 [41] | 5.08 [59] | 8.7 [92] | 5.97 [67] |
| SSKEQSGIPEENEKN | 0 [22] | 7.14 [56] | 7.32 [41] | 8.47 [59] | 4.35 [92] | 5.97 [67] |
| SNQGESGSPEENEKN | 0 [22] | 3.57 [56] | 2.44 [41] | 1.69 [59] | 6.52 [92] | 4.48 [67] |
| SSKEQSCIPEENEKN | 0 [22] | 3.57 [56] | 0 [41] | 1.69 [59] | 0 [92] | 0 [67] |
| SSKEESGSPEENEKN | 0 [22] | 1.79 [56] | 0 [41] | 0 [59] | 0 [92] | 0 [67] |
| SSKEQIGIPDENEKN | 0 [22] | 1.79 [56] | 0 [41] | 0 [59] | 0 [92] | 0 [67] |
| SSKEQSGIPDENEKN | 0 [22] | 1.79 [56] | 7.32 [41] | 3.39 [59] | 5.43 [92] | 4.48 [67] |
| CSKEQSGSPDENEKN | 0 [22] | 0 [56] | 2.44 [41] | 0 [59] | 0 [92] | 0 [67] |
| SNKEQSGSPDENEKN | 0 [22] | 0 [56] | 2.44 [41] | 0 [59] | 0 [92] | 0 [67] |
| SNKEQSGSPEENEKN | 0 [22] | 0 [56] | 2.44 [41] | 0 [59] | 0 [92] | 0 [67] |
| SNQGQSGSSEENEKN | 0 [22] | 0 [56] | 2.44 [41] | 0 [59] | 0 [92] | 0 [67] |
| SSKEESGIPEENEKN | 0 [22] | 0 [56] | 2.44 [41] | 0 [59] | 2.17 [92] | 0 [67] |
| CNQGQSGIPDENEKN | 0 [22] | 0 [56] | 0 [41] | 1.69 [59] | 0 [92] | 1.49 [67] |
| CNQGQSGIPEENEKN | 0 [22] | 0 [56] | 0 [41] | 1.69 [59] | 2.17 [92] | 1.49 [67] |
| CNQGQSGSPEENEKN | 0 [22] | 0 [56] | 0 [41] | 1.69 [59] | 4.35 [92] | 1.49 [67] |
| SNQGQSGSPGENEKN | 0 [22] | 0 [56] | 0 [41] | 1.69 [59] | 0 [92] | 0 [67] |
| SNQGESGIPDENEKN | 0 [22] | 0 [56] | 0 [41] | 0 [59] | 1.09 [92] | 0 [67] |
| SNQGQSGIPEENEKY | 0 [22] | 0 [56] | 0 [41] | 0 [59] | 0 [92] | 2.99 [67] |
| SNQGQNGSPEKNEKN | 0 [22] | 0 [56] | 0 [41] | 0 [59] | 0 [92] | 1.49 [67] |
| SNQGQSGIPDEYEKN | 0 [22] | 0 [56] | 0 [41] | 0 [59] | 0 [92] | 1.49 [67] |
| SNQGQSGIPEKNEKN | 0 [22] | 0 [56] | 0 [41] | 0 [59] | 0 [92] | 1.49 [67] |
| SNQGQSGSPDKNEKN | 0 [22] | 0 [56] | 0 [41] | 0 [59] | 0 [92] | 1.49 [67] |
| SNQGQSGSPEENQKN | 0 [22] | 0 [56] | 0 [41] | 0 [59] | 0 [92] | 1.49 [67] |
| SNQGQSGSPEKNEKN | 0 [22] | 0 [56] | 0 [41] | 0 [59] | 0 [92] | 1.49 [67] |
| SSKEQSGSPEENERN | 0 [22] | 0 [56] | 0 [41] | 0 [59] | 0 [92] | 1.49 [67] |

The following were used to define haplotypes: 15 polymorphic sites, including codons 44, 62, 65, 67, 110, 116, 119, 120, 126, 130, 188, 283, 308, 338 and 409. The haplotype marked with an * indicate the 3D7 haplotype. Frequency is presented as the percentage of sequences that contributed to the haplotype out of the total number of sequences [n]. In grey are zero SNP frequencies.

**Supplementary Table 14.** *serine-tRNA ligase* haplotype frequencies

| Haplotype | Frequency % [n] | | | | |
| --- | --- | --- | --- | --- | --- |
|  | 1995/96 | 1998/99 | 2005/06 | 2012/13 | 2015/16 |
| NDLIEQLVAATAES* | 93.2 [74] | 85.2 [102] | 95.8 [72] | 86.1 [101] | 84.6 [117] |
| NNLIEQLVAATAES | 4.0 [74] | 0 [102] | 0 [72] | 0 [101] | 0 [117] |
| NDVIEQLVAATAES | 2.7 [74] | 3.9 [102] | 4.1 [72] | 1.9 [101] | 2.5 [117] |
| NDLIDQLVAATAES | 0 [74] | 4.9 [102] | 0 [72] | 0.9 [101] | 3.4 [117] |
| NDLIEQLVAATAEA | 0 [74] | 1.9 [102] | 0 [72] | 0 [101] | 0.8 [117] |
| NDLIDQLVAATAKS | 0 [74] | 0.9 [102] | 0 [72] | 0 [101] | 0 [117] |
| NDLIDQLVAATVES | 0 [74] | 0.9 [102] | 0 [72] | 0 [101] | 0 [117] |
| NDLIEQFVAATAES | 0 [74] | 0.9 [102] | 0 [72] | 0 [101] | 0 [117] |
| NDLIEQLVAGTAES | 0 [74] | 0.9 [102] | 0 [72] | 1.9 [101] | 0.8 [117] |
| NDLIEQLVAAPAES | 0 [74] | 0 [102] | 0 [72] | 5.9 [101] | 1.7 [117] |
| NDLIEQLLAATAES | 0 [74] | 0 [102] | 0 [72] | 1.9 [101] | 0 [117] |
| NDLIEELVAATAES | 0 [74] | 0 [102] | 0 [72] | 0.9 [101] | 0.8 [117] |
| SDLIEQLVAATAES | 0 [74] | 0 [102] | 0 [72] | 0 [101] | 3.4 [117] |
| NDLIEQLVVATAES | 0 [74] | 0 [102] | 0 [72] | 0 [101] | 0.8 [117] |
| NDLMEQLVAATAES | 0 [74] | 0 [102] | 0 [72] | 0 [101] | 0.8 [117] |

The following were used to define haplotypes: 14 polymorphic sites, including codons 31, 71, 84, 86, 88, 94, 140, 155, 199, 221, 267, 284, 318 and 417. The haplotype marked with an * indicate the 3D7 haplotype. Frequency is presented as the percentage of sequences that contributed to the haplotype out of the total number of sequences [n]. In grey are zero SNP frequencies.

**Supplementary Table 15.** Spatial temporal variation in alleles between Kilifi North and Kilifi South.

| **Timepoint** | **Region** | ***crt*** | | ***mdr1*** | | | | | | | | ***nfs*** | |
| --- | --- | --- | --- | --- | --- | --- | --- | --- | --- | --- | --- | --- | --- |
|  |  | **CVIET** | **CVMNK** | **NFD** | **NFY** | **NYD** | **NYY** | **YFD** | **YFY** | **YYD** | **YYY** | **K** | **Q** |
| 1995/96 | Kilifi North | 14 | 10 | 4 | 0 | 4 | 0 | 0 | 0 | 3 | 7 | 7 | 14 |
|  | Kilifi South | 14 | 9 | 5 | 0 | 1 | 0 | 0 | 2 | 6 | 5 | 14 | 15 |
| 1998/99 | Kilifi North | 48 | 5 | 4 | 0 | 5 | 1 | 0 | 0 | 8 | 25 | 10 | 31 |
|  | Kilifi South | 38 | 1 | 2 | 4 | 3 | 0 | 0 | 0 | 3 | 18 | 9 | 9 |
| 2005/06 | Kilifi North | 11 | 9 | 1 | 1 | 1 | 0 | 1 | 0 | 1 | 2 | 4 | 8 |
|  | Kilifi South | 14 | 18 | 2 | 0 | 3 | 0 | 0 | 0 | 7 | 5 | 8 | 11 |
| 2012/13 | Kilifi North | 0 | 2 | 0 | 0 | 1 | 0 | 0 | 0 | 0 | 0 | 0 | 4 |
|  | Kilifi South | 4 | 29 | 6 | 0 | 12 | 0 | 0 | 0 | 1 | 0 | 14 | 22 |
| 2015/16 | Kilifi North | 0 | 3 | 5 | 0 | 3 | 0 | 0 | 0 | 0 | 0 | 1 | 4 |
|  | Kilifi South | 1 | 40 | 18 | 0 | 11 | 0 | 0 | 0 | 1 | 0 | 13 | 31 |
| 2016/17 | Kilifi North | 0 | 6 | 2 | 0 | 3 | 0 | 0 | 0 | 0 | 0 | 1 | 4 |
|  | Kilifi South | 1 | 36 | 12 | 1 | 17 | 0 | 0 | 0 | 0 | 0 | 5 | 24 |

The table shows the variation of alleles between Kilifi North and Kilifi South over time for *crt*, *mdr1* and *nfs*. This analysis focussed on the three markers as these showed significant temporal variation in alleles between pre- and post-ACT introduction and have also been validated as drug-resistance markers for chloroquine and ACT partner drug - lumefantrine. Over time, there were increases in the wildtype *crt* and *mdr1* alleles as well as mutant *nfs* alleles but there was no clustering of alleles by Kilifi North vs Kilifi South.

## References

Conrad, M. D., Bigira, V., Kapisi, J., Muhindo, M., Kamya, M. R., Havlir, D. V, … Rosenthal, P. J. (2014). Polymorphisms in *k13* and *falcipain-2* associated with artemisinin resistance are not prevalent in *Plasmodium falciparum* isolated from Ugandan children. *PLoS ONE*, *9*(8), e105690. https://doi.org/10.1371/journal.pone.0105690

Henriques, G., Hallett, R. L., Beshir, K. B., Gadalla, N. B., Johnson, R. E., Burrow, R., … Sutherland, C. J. (2014). Directional Selection at the pfmdr1, pfcrt, pfubp1, and pfap2mu Loci of Plasmodium falciparum in Kenyan Children Treated With ACT. *The Journal of Infectious Diseases*, *210*(12), 2001–2008. https://doi.org/10.1093/infdis/jiu358

Okombo, J., Kamau, A. W., Marsh, K., Sutherland, C. J., & Ochola-Oyier, L. I. (2014). Temporal trends in prevalence of Plasmodium falciparum drug resistance alleles over two decades of changing antimalarial policy in coastal Kenya. *International Journal for Parasitology: Drugs and Drug Resistance*, *4*(3), 152–163. https://doi.org/10.1016/j.ijpddr.2014.07.003
